# Intranasal multivalent adenoviral-vectored vaccine protects against replicating and dormant *M.tb* in conventional and humanized mice

**DOI:** 10.1038/s41541-023-00623-z

**Published:** 2023-02-23

**Authors:** Sam Afkhami, Michael R. D’Agostino, Maryam Vaseghi-Shanjani, Madeleine Lepard, Jack X. Yang, Rocky Lai, Margaret Wa Yan Choi, Alexis Chacon, Anna Zganiacz, Kees L. M. C. Franken, Hildegund C. Ertl, Tom H. M. Ottenhoff, Mangalakumari Jeyanathan, Amy Gillgrass, Zhou Xing

**Affiliations:** 1 McMaster Immunology Research Centre, Department of Medicine, and Michael G. DeGroote Institute for Infectious Disease Research, McMaster University, Hamilton, ON Canada; 2 Leiden University Medical Centre, Leiden, The Netherlands; 3 The Wistar Institute, Philadelphia, PA USA

**Keywords:** DNA vaccines, Tuberculosis

## Abstract

Viral-vectored vaccines are highly amenable for respiratory mucosal delivery as a means of inducing much-needed mucosal immunity at the point of pathogen entry. Unfortunately, current monovalent viral-vectored tuberculosis (TB) vaccine candidates have failed to demonstrate satisfactory clinical protective efficacy. As such, there is a need to develop next-generation viral-vectored TB vaccine strategies which incorporate both vaccine antigen design and delivery route. In this study, we have developed a trivalent chimpanzee adenoviral-vectored vaccine to provide protective immunity against pulmonary TB through targeting antigens linked to the three different growth phases (acute/chronic/dormancy) of *Mycobacterium tuberculosis* (*M.tb*) by expressing an acute replication-associated antigen, Ag85A, a chronically expressed virulence-associated antigen, TB10.4, and a dormancy/resuscitation-associated antigen, RpfB. Single-dose respiratory mucosal immunization with our trivalent vaccine induced robust, sustained tissue-resident multifunctional CD4^+^ and CD8^+^ T-cell responses within the lung tissues and airways, which were further quantitatively and qualitatively improved following boosting of subcutaneously BCG-primed hosts. Prophylactic and therapeutic immunization with this multivalent trivalent vaccine in conventional BALB/c mice provided significant protection against not only actively replicating *M.tb* bacilli but also dormant, non-replicating persisters. Importantly, when used as a booster, it also provided marked protection in the highly susceptible C3HeB/FeJ mice, and a single respiratory mucosal inoculation was capable of significant protection in a humanized mouse model. Our findings indicate the great potential of this next-generation TB vaccine strategy and support its further clinical development for both prophylactic and therapeutic applications.

## Introduction

Pulmonary tuberculosis (TB) remains a leading cause of global morbidity and mortality by a single infectious agent, accounting for 10 million cases and 1.5 million deaths annually with an estimated 1/4 world population being latently infected^1^. The END TB initiative led by the World Health Organization remains an integral strategy in combatting TB, in part relying on enhanced disease surveillance, treatment access, and connected health networks in endemic regions. Although this initiative has globally had a positive impact in TB control, the emergence and continued spread of SARS-CoV-2 has reversed years of progress, as 2020 represented the first year with increased incidences of TB disease and death^1,2^. Despite TB being a global phenomenon, its highest burden is seen in TB-endemic regions that were heavily impacted by the COVID-19 pandemic. These findings highlight the importance of developing and refining strategies that can combat TB without the continuous dependence on lifelines that can be rapidly derailed.

Vaccination aims to generate long-lasting host immunity and is a critical pillar in global TB control and its ultimate elimination. Unfortunately, the only approved TB vaccine, Bacillus Calmette-Guerin (BCG), which has globally been administered intradermally, shortly after birth for more than 7 decades, has failed to provide effective protection against adult pulmonary TB^3,4^. Over the last couple of decades, there has been an intense global effort in developing novel vaccine strategies to boost BCG-primed immunity. In this regard, currently there are at least a dozen lead TB vaccine candidates at various stages of clinical evaluation^5,6^. Among these current-generation vaccines are the two candidates (viral-vectored MVA85A, and subunit-based M72/AS01_E_) that have recently been evaluated for their protective efficacy in phase 2b trials. Unfortunately, these trials either showed no enhancement in protection (MVA85A) or demonstrated only partial efficacy in a selected cohort of participants (M72/ASO1_E_)^7,8^. Since several of the lead vaccine candidates currently in the pipeline are similar to these two candidates in vaccine design, it is uncertain that they will ultimately be successful. Of note, the vast majority of current-generation recombinant subunit and viral-vectored TB vaccines are limited in vaccine antigen coverage and are administered intradermally or intramuscularly. One powerful way to improve vaccine efficacy is to deliver the vaccine via the respiratory route for induction of robust protective respiratory mucosal immunity^4,9^. In this regard, we and others have developed viral-vectored TB vaccines for respiratory route of delivery and have provided supporting clinical evidence for their safety and immunogenicity following aerosol delivery to human lungs^10–12^. However, these viral-vectored vaccines were designed to express only a single-stage *M.tb* antigen, Ag85A, produced mostly during the acute stage of infection^6,13,14^ similar to several other subunit or viral-vectored TB vaccine candidates^5,15,16^. This reality calls for the development of next-generation multivalent viral-vectored TB vaccines that incorporate both the *M.tb* life-cycle spectrum antigens and route of vaccine delivery into the design of vaccine strategy.

Indeed, it is increasingly recognized that next-generation vaccine strategies need to consider the life cycle of *M.tb* bacilli during infection^6,13,17^. Upon infection, confronting the host’s immune pressure and environmental stresses such as nutrient deprivation and oxygen depletion^18–20^, *M.tb* bacilli metabolically shift from an actively replicating state to a quiescent state of persistence, becoming non-replicating persisters or dormant *M.tb* bacilli, a feature of latent TB. This process is associated with differential *M.tb* antigen (Ag) expression with some Ags such as Ag85 complex proteins (AgA/B/C) predominantly produced during the acute stage of infection, some including ESX secretion system proteins (TB10.4, ESAT6 etc.) produced throughout the course of infection, and some expressed during either the latency/dormancy or resusciation^6,13,17,21,22^. Infection stage-dependent Ag expression represents one of the immune-evasive strategies for *M.tb* and can render the current vaccines expressing only acute-stage Ags ineffective, particularly in host defense against chronic and latent TB^23^. Since the front-line TB antibiotics can only target the actively replicating *M.tb* bacilli, the latent TB and its reactivation represent the greatest challenge to TB control and vaccine development. Thus, it is believed that a well-designed next-generation vaccine ought to be multivalent, expressing and targeting the *M.tb* Ags produced throughout its entire life cycle^22,24–27^.

Among the *M.tb* Ags antigens expressed during dormancy are the five resuscitation-promoting factors (rpfA/B/C/D/E) involved in the resuscitation of dormant *M.tb* bacilli and TB reactivation^17,21^. Humans with latent TB were found to harbor the T cells strongly reactive to rpf antigens^28^. Preclinical studies have further revealed that RpfB Ag is most immunogenic of the five rpf Ags and represents a robust CD8^+^ T-cell activator^29–31^. Although some of the dormancy-associated Ags including select rpf members have been included in the design of recombinant subunit or viral-vectored multistage vaccines^24–27,32–34^, whether such vaccines have any impact on non-replicating dormant *M.tb* bacilli still remains unknown. This is due to the fact that the conventional method to determine the TB vaccine protective efficacy is via enumerating colony-forming unit (CFU) of replicating bacilli cultured on solid agar which does not capture the non-replicating dormant *M.tb*^13,35^. Furthermore, the multivalent TB vaccine design has not been incorporated into the respiratory mucosal immunization strategy with recombinant viral vectors.

We have preclinically and clinically demonstrated that recombinant adenoviral (Ad)-vectored TB vaccines are particularly effective for respiratory mucosal immunization, being able to induce long-lasting tissue-resident trained innate and adaptive immunity^4,10,36–38^. Compared to its human Ad5 (AdHu5)-vectored counterpart, our previous work has also shown that respiratory delivery of a monovalent chimpanzee AdCh68-vectored TB vaccine (Mono:ChAd:TB) was even more immunogenic and could provide robust prophylactic and therapeutic protection^39,40^. The advantage of the AdCh68 vector over the AdHu5 backbone is further supported by our recent report on Ad-vectored COVID-19 vaccine strategies^41^. Hence, our current study set out to develop and evaluate a next-generation multivalent AdCh68-vectored TB vaccine for respiratory mucosal immunization. This vaccine was designed to target the antigens expressed at the different growth phases of *M.tb* (acute/chronic/dormancy) by expressing the three select *M.tb* Ags, Ag85A, TB10.4, and RpfB, whose expression profiles have been well-described in vivo and in vitro, for induction of broadly protective immunity against pulmonary TB^42^. We show that respiratory mucosal vaccination with this multivalent vaccine provides remarkable protection against two virulent strains of *M.tb* in three separate murine models including a humanized mouse model. Of importance, for the first time, we provide evidence that this vaccine is able to expand protective efficacy by controlling both replicating and non-replicating dormant *M.tb* bacilli in the lung. We further show that this vaccine strategy could accelerate TB control adjunct to TB antibiotic therapy.

## Results

### Molecular construction and characterization of a multivalent ChAd:TB vaccine

Stochastic and immune-mediated changes in the antigenic profile of *M.tb* contribute to enhanced evasion of host adaptive immune responses^23^. As such, there is interest in expanding the antigenic breadth of TB vaccine candidates to better encompass its multivalent life cycle^24,26,43^. We have previously shown that intranasal (i.n.) vaccination with a monovalent ChAd-vectored vaccine expressing the acute-stage antigen, antigen 85A (Ag85A) (herein referred to as Mono:ChAd:TB) provides robust protection against pulmonary TB^39,40^. To further address the importance of expanding vaccine-mediated immunity against the different stages of the *M.tb* life cycle (acute/chronic/dormancy)^42^, we re-engineered our platform to include two additional antigens, TB10.4, and RpfB (herein referred to as Tri:ChAd:TB). Antigen 85A and TB10.4 are well-characterized, potent immunogens which are recognized by T cells in infected individuals and have been experimentally shown to confer immunity against TB^44–46^. However, in comparison to Ag85A whose expression profile is significantly reduced following the acute stage of infection, TB10.4 expression remains stably high^43,47,48^. In contrast, RpfB is not only expressed during dormancy, but its expression is critical in mycobacterial resuscitation as the bacilli transition between a state of persistence and active replication, as characterized in in vitro and in vivo murine model systems^49^. As such, targeting immune responses to RpfB allows for directing vaccine immunity against such persistent, difficult-to-eliminate subpopulations^31,49–53^.

**Fig. 1:**
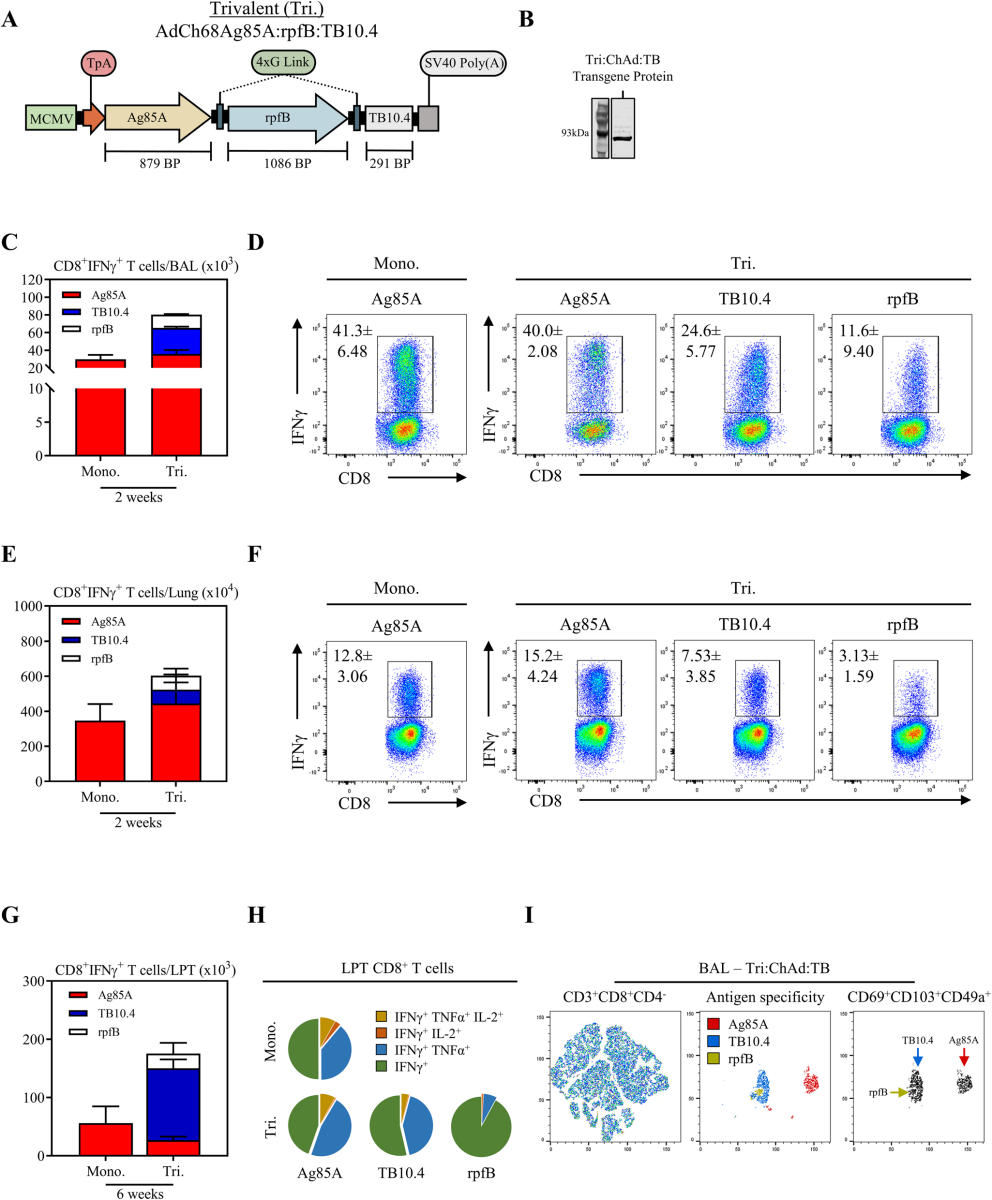
Transgene design and immunogenicity of a multivalent ChAd:TB vaccine. **A** Transgene cassette diagram for Tri:ChAd:TB. **B** Left: Transgene expression analysis by western blot using protein isolated from A549 cells infected with Tri:ChAd:TB. Fusion protein derived from transgene expression was probed with an anti-TB10.4 monoclonal antibody. **C** Stacked bar graphs depicting absolute numbers of CD8^+^IFNγ^+^ T-cell responses in the BAL 2 weeks post-intranasal (i.n.) vaccination with either Mono:ChAd:TB or Tri:ChAd:TB, as measured by expression of IFNγ following ex vivo stimulation with Ag85A (red), TB10.4 (blue), or RpfB (white) whole protein. **D** Representative flow cytometric plots of IFNγ^+^CD8^+^ T cells in the BAL 2 weeks post-i.n. immunization with either Mono:ChAd:TB or Tri:ChAd:TB, following ex vivo stimulation with Ag85A, TB10.4, or RpfB whole protein. Gating strategy provided in Supplemental Fig. 5. **E** Stacked bar graphs depicting absolute numbers of CD8^+^IFNγ^+^ T-cell responses in the lung 2 weeks post-i.n. immunization with either Mono:ChAd:TB or Tri:ChAd:TB, as measured by expression of IFNγ following ex vivo stimulation with Ag85A (red), TB10.4 (blue), or RpfB (white) whole protein. **F** Representative flow cytometric plots of IFNγ^+^CD8^+^ T cells in the lung 2 weeks post-i.n. immunization with either Mono:ChAd:TB or Tri:ChAd:TB, following ex vivo stimulation with Ag85A, TB10.4, or RpfB whole protein. **G** Stacked bar graphs depicting absolute numbers of CD8^+^IFNγ^+^ T-cell responses in the lung parenchymal tissue (LPT) 6 weeks post-i.n. immunization with either Mono:ChAd:TB or Tri:ChAd:TB, as measured by expression of IFNγ following ex vivo stimulation with Ag85A (red), TB10.4 (blue), or RpfB (white) whole protein. **H** Pie charts depicting the functionality (IFNγ, TNFα, and/or IL-2) of LPT CD8^+^ T cells 6 weeks post-i.n. immunization with either Mono:ChAd:TB or Tri:ChAd:TB, following ex vivo stimulation with Ag85A, TB10.4, or RpfB whole protein. **I** Left panel: t-SNE map generated from concatenating CD3^+^CD8^+^CD4^−^ gated cells from lung mononuclear cells from animals i.n.-vaccinated with tri:ChAd:TB and stimulated with either Ag85A,TB10.4, or RpfB whole protein. Middle panel: Overlayed populations representing either Ag85A (red), TB10.4 (blue), or RpfB (green)-specific T cells. Right panel: Heatmap projection of CD69^+^CD103^+^CD49a^+^ populations. Data presented in (C–G) represent mean ± SEM of *n* = 3 mice/group. Data are representative of one independent experiment.

The Tri:ChAd:TB vaccine was molecularly constructed utilizing the chimpanzee adenovirus serotype 68 backbone through a direct-cloning method^54^. The transgene cassette was designed to express *Ag85A*, *TB10.4*, and *rpfB*, as a single transcript, with transgene expression under the control of the murine cytomegalovirus (MCMV) promoter, and transgene protein secretion rendered by a human tissue plasminogen signal peptide sequence (TpA) (Fig. 1A). *Ag85A* and *TB10.4* were cloned to flank *rpfB* with the use of flexible glycine (gly) linkers. This design approach therefore allows for the expression of all three antigens linked together as a single polypeptide. Prior to viral rescue, the transgene cassette was sequence verified by Sanger sequencing. Tri:ChAd:TB was rescued through packaging and propagation in HeK 293 cells and purified by cesium chloride banding.

To further characterize this vaccine, A549 cells were infected with Tri:ChAd:TB and cellular lysates were collected for protein isolation. Transgene expression was verified through western blot analysis, indicating a single protein band of ~90 kDa size from the transgene cassette expressed by Tri:ChAd:TB (Fig. 1B).

Following rescue and transgene assessment of Tri:ChAd:TB, we next characterized the acute safety profile of this vaccine following i.n. immunization in the murine model (Supplemental Fig. 1A). In line with our previous studies utilizing the same chimpanzee adenoviral vector, we observed no acute clinical safety signals^39–41^. Assessment of lung tissues by hematoxylin and eosin staining indicated minimal tissue inflammation consisting of mild cellular infiltration around the conducting airways in comparison to naive controls (Supplemental Fig. 1B). Immune profiling of airway cells isolated by bronchoalveolar lavage (BAL) showed small frequencies of neutrophils but few monocytes within the airways in comparison to naive controls (Supplemental Fig. 1C). Collectively, our data support the favorable in vivo safety profile of Tri:ChAd:TB vaccination.

### Intranasal immunization with a multivalent ChAd:TB vaccine induces antigen-specific T-cell responses in the airways and lung tissues

We first characterized the T-cell immunogenicity of Tri:ChAd:TB following respiratory mucosal immunization. Mice were vaccinated intranasally (i.n.) with a single dose of Tri:ChAd:TB (Tri). For comparison, a group of mice were i.n. immunized with an equal dose of its monovalent counterpart, Mono:ChAd:TB (Mono). T-cell responses were analyzed in the airway lumen, represented by bronchoalveolar lavage (BAL) and lung tissue 2 weeks post immunization. Vaccine antigen-specific T-cell responses were quantified by intracellular cytokine staining (IFNγ^+^) and flow cytometry following ex vivo stimulation with either recombinant Ag85A, TB10.4, or RpfB proteins. Intranasal Tri:ChAd:TB immunization induced high levels of Ag85A-, TB10.4-, and RpfB-specific CD8^+^ T cells in the airway lumen (Fig. 1C, D) and lung tissue (Fig. 1E, F). Notably, Ag85A-specific CD8^+^ T cells were greater in magnitude compared to TB10.4- and RpfB-specific CD8^+^ T cells both in the BAL and lung. Importantly, Ag85A-specific responses induced by Tri:ChAd:TB were comparable to the levels of Ag85A-specific responses induced by monovalent counterpart, indicating no antigenic competition despite inclusion of two additional antigens in Tri:ChAd:TB. Intranasal Tri:ChAd:TB immunization also induced Ag85A-, TB10.4-, and RpfB-specific CD4^+^IFNγ^+^ T cells in the airway lumen (Supplemental Fig. 2A, B) and lung tissue (Supplemental Fig. 2C, D), but to a lesser degree than CD8^+^ T-cell responses. These data suggest that our multivalent ChAd:TB vaccine is capable of inducing robust T-cell responses at the respiratory mucosal surface and in the lung against all three vaccine-encoded antigens.

### Intranasal immunization with a multivalent ChAd:TB vaccine induces multifunctional tissue-resident memory T cells

Since long-lasting multifunctional-tissue-resident (T_RM_) Ag-specific T cells at respiratory mucosal surfaces induced following vaccination are critical for effective host defense against pulmonary TB^40,55–57^, we next evaluated the longevity, functionality and phenotypic characteristics of Ag-specific T cells induced following Tri:ChAd:TB vaccination and compared that to Ag-specific T cells induced by its monovalent counterpart. Mice were i.n. vaccinated with either Tri:ChAd:TB or Mono:ChAd:TB and T-cell responses in the airway lumen (BAL) and lung parenchymal tissue (LPT) were assessed 6 weeks post immunization. Bona fide T cells within LPT were differentiated from intravascular counterparts (LV) via intravascular CD45.2 immunolabelling (Supplemental Fig. 2E)^58^. To profile the multifunctionality of CD8^+^ T-cell responses, total BAL and lung mononuclear cells were ex vivo stimulated with congruent recombinant proteins as described above and subjected to intracellular cytokines staining for IFNγ, TNFα, and IL-2. A sizeable population of Ag85A−, TB10.4−, and RpfB-specific CD8^+^IFNγ^+^ T cells still remained in the LPT 6 weeks post-Tri:ChAd:TB immunization (Fig. 1G). Interestingly, proportions of Ag85A-, TB10.4- and RpfB-specific CD8^+^IFNγ^+^ T cells at 6 weeks post immunization differed considerably from the proportions at 2 weeks post immunization. While Ag85A-specific CD8^+^IFNγ^+^ T cells predominated during the effector phase (Fig. 1E), TB10.4-specific CD8^+^IFNγ^+^ T cells predominated during the memory phase (Fig. 1G). In keeping with effector phase, the magnitude of Ag85A-specific CD8^+^IFNγ^+^ T-cell responses were comparable between Tri:ChAd:TB and Mono:ChAd:TB-immunized hosts during the memory phase (Fig. 1G). Functionally, the majority of Ag85A- and TB10.4-specific CD8^+^ T cells were bi-(IFNγ^+^ TNFα^+^) or mono-(IFNγ^+^) functional in Tri:ChAd:TB-immunized hosts (Fig. 1H). In contrast RpfB-specific CD8^+^ T cells were primarily monofunctional, solely producing IFNγ. Ag85A-specific CD8^+^ T cells induced by Tri:ChAd:TB and Mono:ChAd:TB were functionally similar (Fig. 1H).

Having established that i.n. Tri:ChAd:TB induces long-lasting multifunctional antigen-specific CD8^+^ T cells within the LPT, we next profiled the expression of tissue-resident memory surface markers CD69, CD103, and CD49a^59,60^ by Ag85A-, TB10.4- and RpfB-specific CD8^+^IFNγ^+^ T cells using t-SNE analysis on concatenated CD3^+^CD8^+^CD4^−^ BAL mononuclear cells from Tri:ChAd:TB-immunized animals (Fig. 1I, left panel). Within the t-SNE map of total BAL CD8^+^ T cells, overlayed congruent Ag-specific CD8^+^ T cells clustered into three unique populations representing Ag85A-, TB10.4-, and RpfB-specific cells (Fig. 1I, middle panel). Expression of resident markers CD69, CD103 and CD49a by t-SNE analysis identified that the majority of Ag-specific cells being tissue-resident memory CD8^+^ T cells (Fig. 1I, right panel).

Collectively, the above data suggest that vaccine antigen-specific CD8^+^ T cells induced by i.n. Tri:ChAd:TB immunization are sustained at the mucosal surfaces, are multifunctional, and acquire a bona fide lung resident memory phenotype.

### Intranasal vaccination with a multivalent ChAd:TB vaccine markedly boosts antigen-specific T-cell responses in parenteral BCG-primed hosts

**Fig. 2:**
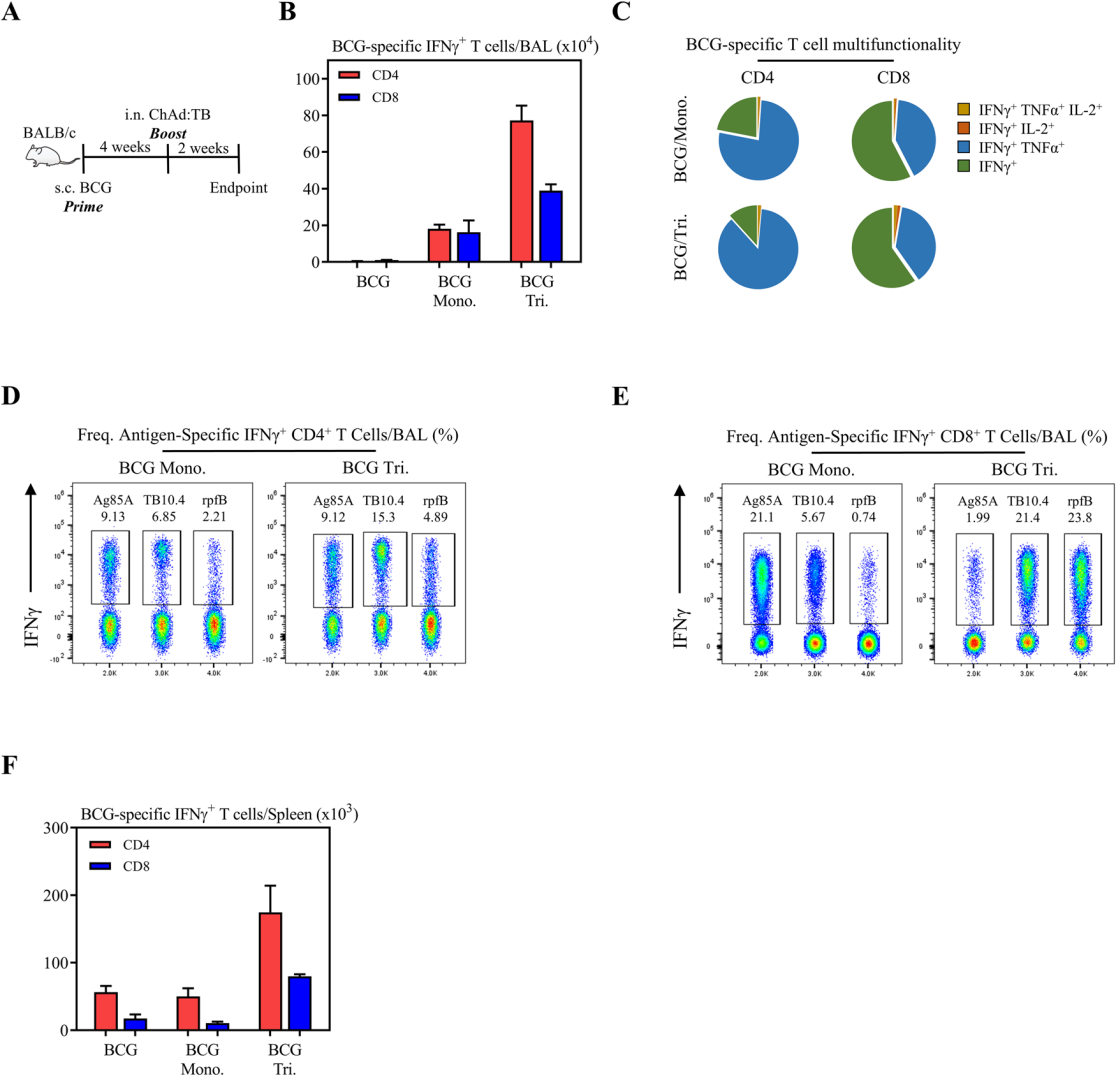
Immunogenicity of a multivalent ChAd:TB vaccine in BCG-primed animals. **A** Experimental schema. **B** Bar graphs depicting absolute numbers of either CD4^+^ (red) or CD8^+^ (blue) T-cell responses in the BAL, as measured by expression of IFNγ following ex vivo stimulation with crude BCG/culture filtrate. **C** Pie charts depicting the functionality (IFNγ, TNFα, and/or IL-2) of CD8^+^ or CD4^+^ T cells in the BAL following ex vivo stimulation with crude BCG/culture filtrate. **D** Flow cytometric plots of IFNγ^+^CD4^+^ T cells in the BAL from concatenating CD3^+^ gated cells following ex vivo stimulation with Ag85A, TB10.4, or RpfB whole protein. **E** Flow cytometric plots of IFNγ^+^CD8^+^ T cells in the BAL from concatenating CD3^+^ gated cells following ex vivo stimulation with Ag85A, TB10.4, or RpfB whole protein. **F** Bar graphs depicting absolute numbers of either CD4^+^ (red) or CD8^+^ (blue) T-cell responses in the spleen, as measured by expression of IFNγ following ex vivo stimulation with crude BCG/culture filtrate. Data presented in (**B**, **F**) represent mean ± SEM of *n* = 3 mice/group. Data are representative of one independent experiment.

Despite being less efficacious against adult pulmonary forms of TB, the effectiveness of BCG against disseminated childhood disease makes it a foundation of the global immunization program for TB. As such, next-generation TB vaccines should aim to boost protective immunity in BCG-primed humans. Given this, we next sought to examine the boosting efficacy of Tri:ChAd:TB. To this end, mice were either subcutaneously (s.c.) immunized with BCG alone (BCG), or were subsequently i.n. boosted 4 weeks post-BCG with Tri:ChAd:TB (BCG Tri.). A set of BCG-primed mice were i.n. boosted with Mono:ChAd:TB (BCG Mono.) as a comparison. Animals were sacrificed 2 weeks post boost and mononuclear cells were isolated from the airway lumen (BAL) and lung tissue (Fig. 2A). Antigen-specific CD4^+^ and CD8^+^ T cells were quantified by flow cytometry for expression of IFNγ following ex vivo stimulation with either crude BCG antigens, or recombinant Ag85A, TB10.4, or RpfB proteins. Consistent with our previous findings^40,61^, parenteral BCG priming alone did not induce airway luminal CD4^+^ and CD8^+^ T-cell responses (Fig. 2B), whereas it induced a level of BCG antigen reactive CD4^+^ and CD8^+^ T-cell responses (BCG-specific) in the lung tissue (Supplemental Fig. 3A). In keeping with our previous findings, i.n. Mono:ChAd:TB boost immunization of BCG-primed hosts (BCG Mono.) markedly increased BCG-specific CD4^+^ T cells in the airway lumen (BAL) and lung (Fig. 2B and Supplemental Fig. 3A)^40^. It also increased BCG-specific CD8^+^ T cells in the BAL (Fig. 2B). In comparison, Tri:ChAd:TB respiratory mucosal boosting markedly increased both CD4^+^ and CD8^+^ T-cell responses in the airway lumen and lung and the magnitude of such responses were significantly greater than that induced by its monovalent counterpart (Fig. 2B and Supplemental Fig. 3A). Functionality of airway luminal and lung BCG-specific CD4^+^ and CD8^+^ T cells induced by either vaccine in BCG-primed hosts was comparable (Fig. 2C and Supplemental Fig. 3B). Of interest, the majority of airway luminal and lung BCG-specific CD4^+^ T cells were bifunctional (IFNγ^+^ TNFα^+^), whereas BCG-specific CD8^+^ T cells were either monofunctional (IFNγ^+^) or bifunctional (IFNγ^+^TNFα^+^) (Fig. 2C and Supplemental Fig. 3B). Moreover, i.n. boosting with either vaccine markedly increased bifunctional (IFNγ^+^TNFα^+^) BCG-specific CD4^+^ T cells in the lung of BCG-primed hosts (Supplemental Fig. 3B).

Given that Ag85A, TB10.4, and RpfB-specific T-cell responses were detected in BCG-vaccinated hosts^45,46,62^, we next assessed whether these responses were boosted in the airway lumen (BAL), lung, and spleen of BCG-primed hosts following i.n. boosting with Tri:ChAd:TB (BCG/Tri). A set of BCG-primed animals were i.n. immunized with Mono:ChAd:TB (BCG/Mono.) as a comparison (Fig. 2A). Of note, s.c. BCG priming alone did not induce airway luminal CD4^+^ and CD8^+^ T-cell responses (Fig. 2B). Intranasal boosting of BCG-primed hosts with either Tri:ChAd:TB or the monovalent counterpart markedly increased CD4^+^ and CD8^+^ T cells specific to all three antigens in the airway lumen (Fig. 2D, E). Tri:ChAd:TB immunization differed significantly from its monovalent counterpart in its capacity to markedly boost TB10.4 and RpfB-specific CD4^+^ and CD8^+^ T cells in BCG-primed hosts, with a twofold increase in TB10.4 and RpfB-specific CD4^+^ T cells and 4- and 20-fold increase in TB10.4 and RpfB-specific CD8^+^ T cells, respectively, compared to those boosted with its monovalent counterpart (Fig. 2D, E). Mono:ChAd:TB induced significantly increased Ag85A-specific CD8^+^ T cells than Tri:ChAd:TB in the airway lumen (Fig. 2E). Similar trends were observed in the lung (Supplemental Fig. 3C, D). Of interest, i.n. Tri:ChAd:TB immunization in parenteral BCG-primed host not only boosted the respiratory mucosal BCG-specific responses, but also boosted such responses remotely in the spleen (Fig. 2F). This boosting effect was not evident following i.n. boosting with a monovalent counterpart.

The above data together indicate that Tri:ChAd:TB boosting potently enhances BCG- as well as multi-antigen-specific CD4^+^ and CD8^+^ T-cell responses at the respiratory mucosal surfaces and at peripheral sites.

### Intranasal immunization with a multivalent ChAd:TB vaccine provides enhanced protection against pulmonary *M.tb* infection over its monovalent counterpart

**Fig. 3:**
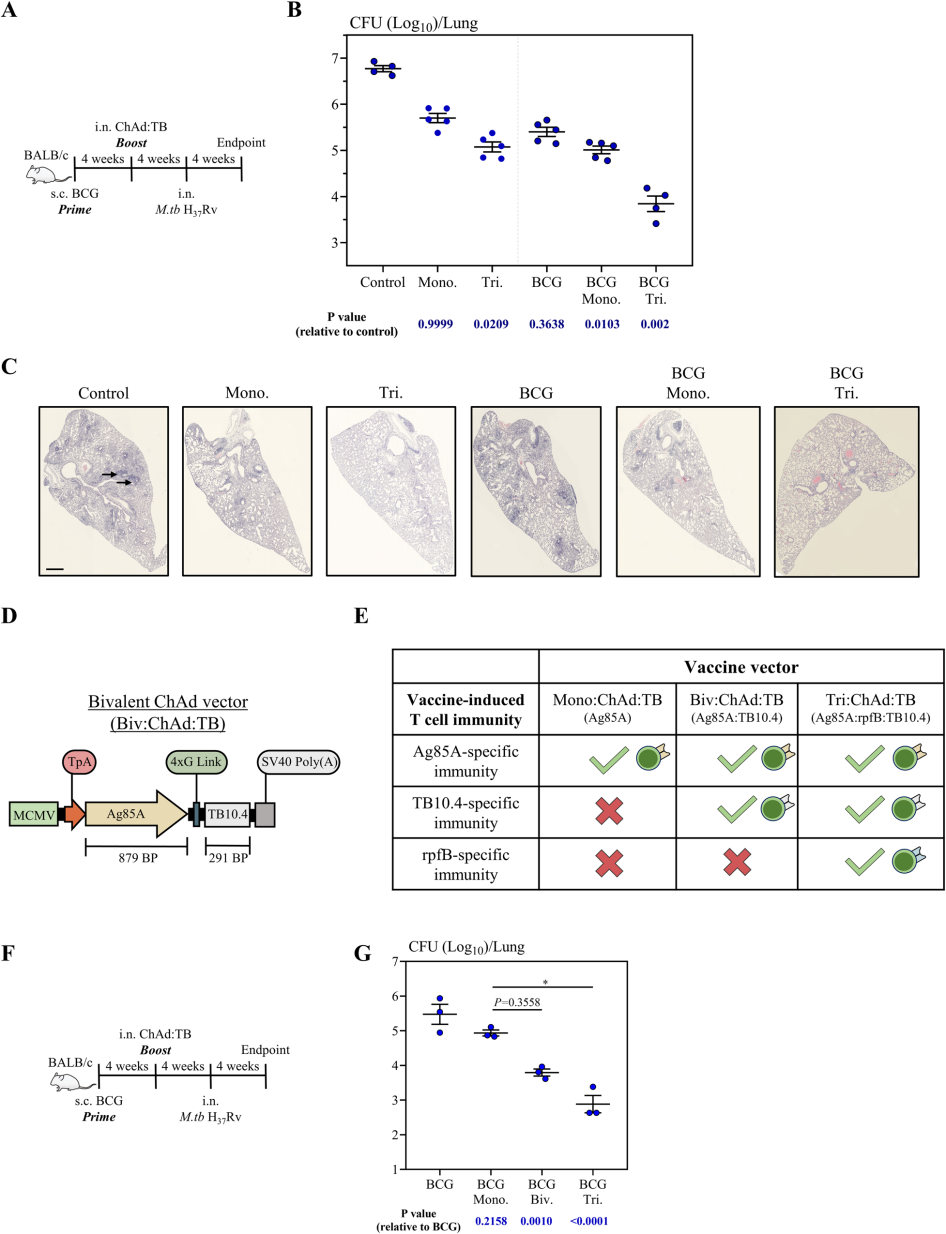
Protective efficacy of a multivalent ChAd:TB vaccine against *M.tb* (H_37_Rv) in the BALB/c model. **A** Experimental schema, pertaining to panels **B** and **C**. **B** Lung mycobacterial burden (Log_10_ colony-forming unit (CFU)) 4 weeks post-*M.tb* (H_37_Rv) challenge via the respiratory mucosal route. **C** Representative lung H&E images 4 weeks post-*M.tb* challenge. Black arrows indicate granulomatous lesions. Scale bars represent 200 µm. **D** Transgene cassette diagram for Biv:ChAd:TB. **E** Conceptual chart depicting the relationship of antigen-specific (Ag85A, TB10.4, and/or RpfB) T-cell immunity to immunization with either Mono:ChAd:TB, Biv:ChAd:TB, or Tri:ChAd:TB. **F** Experimental schema, pertaining to panel **G**. **G** Lung mycobacterial burden (Log_10_ CFU) 4 weeks post-*M.tb* challenge via the respiratory mucosal route. Data presented in (**B**, **G**) represent mean ± SEM of *n* = 3–5 mice/group. Data are representative of one independent experiment. Data in (**B**, **G**) are from two separate independent experiments. Statistical analysis for (**B**, **G**) was performed using the nonparametric Kruskal–Wallis test with Dunn’s multiple comparison test. *P = 0.05.

To investigate whether the induction of multi-antigen-specific responses at the respiratory mucosa in naive and s.c. BCG-primed hosts could lead to improved protection against pulmonary TB, naive mice or 4-week s.c. BCG-primed mice were i.n. immunized with either Tri:ChAd:TB or its monovalent counterpart. As controls, a group of mice was left unvaccinated (control) or s.c. immunized with BCG for 8 weeks. All mice were infected via the respiratory mucosal route with virulent *M.tb* (H_37_Rv), and sacrificed 4 weeks post infection (Fig. 3A). Relative levels of protective efficacy in the lung were assessed by enumerating mycobacterial burden using a solid agar colony-forming unit (CFU) assay. Formalin-fixed lung sections were subjected to hematoxylin and eosin (H&E) staining and histopathological analysis. In agreement with our previous findings, BCG priming (BCG) and i.n. Mono:ChAd:TB (Mono.) as a stand-alone or BCG Mono:ChAd:TB (BCG Mono.) immunization enhanced protection, leading to a 1.0, 1.3, and 1.8log_10_ *M.tb* colony-forming unit (CFU) reduction, respectively, compared to control (Fig. 3B)^40,61^. Of interest, correlating with its ability to induce multi-antigen-specific T-cell immunity in naive hosts (Tri.) (Fig. 1) and markedly boosting both BCG-specific CD4^+^ and CD8^+^ T cells that were also specific to Ag85A, TB10.4, and RpfB (BCG Tri.) (Fig. 2A–D), i.n. Tri:ChAd:TB immunization further improved protection, leading to a 1.7 log_10_ and 2.9 log_10_ reduction in *M.tb* CFU, respectively (Fig. 3B). Protection rendered by immunization with Tri:ChAd:TB alone or boosting BCG-induced immunity were markedly greater than the protection rendered by its monovalent counterpart, causing further reduction in bacterial burden by 4- and 13-fold, respectively.

In keeping with markedly improved protection rendered by Tri:ChAd:TB either as a stand-alone (Tri.) or BCG booster (BCG Tri.) immunization, we observed remarkably reduced lung immunopathology in these animals (Fig. 3C). Although Mono:ChAd:TB either as a stand-alone (Mono.) or BCG booster (BCG Mono.) reduced overall immunopathology compared to unvaccinated (Control) and BCG immunized (BCG) hosts, it did not prevent the occurrence of small focal areas of granulomatous inflammation (black arrows). In stark contrast, naive and BCG-primed hosts that received single dose of i.n. Tri:ChAd:TB displayed only mild inflammatory infiltrates located in the parabronchial and perivascular regions (Fig. 3C). Collectively, the above data indicate that the inclusion of additional antigens (TB10.4 and RpfB) into our trivalent ChAd-vectored vaccine design further improves its protective capacity against pulmonary tuberculosis.

### Inclusion of multiple antigens in vaccine design enhances protection against pulmonary *M.tb* in parenteral BCG-primed hosts

We thus far have demonstrated that our trivalent ChAd:TB vaccine expressing Ag85A, TB10.4, and rpfB confers superior protection relative to its monovalent counterpart expressing Ag85A alone. To further understand how the inclusion of additional antigens from the *M.tb* life cycle enhance protection against pulmonary *M.tb* infection, we generated a bivalent ChAd:TB vaccine expressing Ag85A and TB10.4, herein referred to as Biv:ChAd:TB (Fig. 3D). We profiled this vaccine’s immunogenicity by carrying out similar experiments as Fig. 1G, H, confirming that i.n. Biv:ChAd:TB induces Ag85A- and TB10.4-specific T-cell responses that are qualitatively and quantitatively similar to Tri:ChAd:TB (Supplemental Fig. 4).

Comparing protection conferred by Mono:ChAd:TB to Biv:ChAd:TB allows us to address the protective contribution of TB10.4-specific immunity whereas comparing protection by Biv:ChAd:TB to Tri:ChAd:TB allows us to address the protective contribution of RpfB-specific immunity (Fig. 3E). In this regard, 4 weeks s.c. BCG-primed animals were i.n. immunized with an equal dose of either Mono:ChAd:TB, Biv:ChAd:TB, or Tri:ChAd:TB. A group of mice were s.c. immunized with BCG for 8 weeks. All groups of mice were infected via the respiratory mucosal route with virulent *M.tb* (H_37_Rv) and sacrificed 4 weeks post-challenge (Fig. 3F).

First, we observed successive reduction in pulmonary mycobacterial burden through the inclusion of additional antigens into our vaccine design, with Tri:ChAd:TB-boosted animals being significantly greater protected than Mono:ChAd:TB and Biv:ChAd:TB-boosted animals, and Biv:ChAd:TB-boosted animals being greater protected than Mono:ChAd:TB-boosted animals (Fig. 3G). The greater protection observed following boosting with Biv:ChAd:TB in comparison to Mono:ChAd:TB suggests that inclusion of TB10.4 further enhances the protection conferred by Ag85A-specific immunity alone. In line with these observations, the further enhanced protection observed following boosting with Tri:ChAd:TB in comparison to Biv:ChAd:TB also suggests that inclusion of RpfB further enhances the protection conferred by Ag85A- and TB10.4-specific immunity. Although based on the data presented we cannot conclude about the direct protective contribution of TB10.4 and RpfB-specific immunity alone, the comparison between our Mono., Biv., and Tri:ChAd:TB vectors strongly suggests that expression of additional antigens from the *M.tb* life cycle and thus, the induction of immunity against these antigens (Fig. 2), directly correlate with enhanced protection, with Tri:ChAd:TB vaccine design providing the best level of protection.

### Inclusion of *rpfB* in vaccine design significantly reduces nonculturable, persistent *M.tb* bacilli following antibiotic cessation

Nutrient-supplemented solid agar or liquid media remains the gold standard for assessing mycobacterial burden. However, this method fails to capture mycobacterial subpopulations that do not grow under these conditions. Nonculturable or persistent mycobacterial subpopulations (herein referred to as persisters) arise both stochastically and due to immune and pharmacological pressures^63–65^. Persisters are an occult subpopulation that directly contributes to both the length of antibiotics required for successful disease treatment and concordantly also treatment default and disease relapse^64^. Such populations however can be quantified by supplementing liquid medium cultures with mycobacterial culture filtrates that are rich in factors which promote resuscitation of persisters into an actively replicating state^50,64–67^. Despite the critical importance of persisters in TB, all TB vaccine studies to-date have assessed vaccine efficacy by exclusively quantifying actively replicating mycobacteria by conventional microbiological methods. Therefore, complementing this approach by directly measuring persisters as described above would provide a more accurate assessment of vaccine efficacy.

**Fig. 4:**
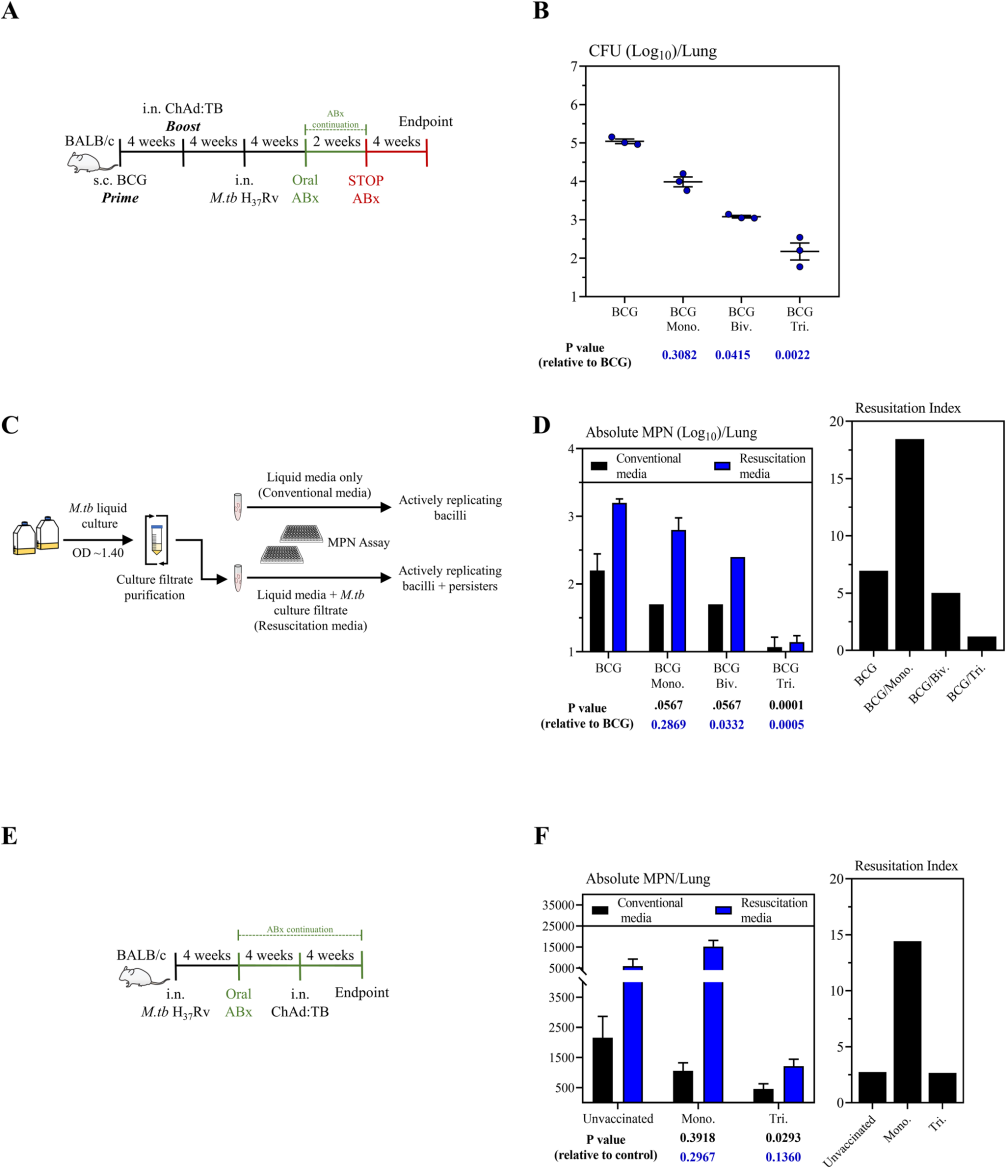
Protective efficacy of a multivalent ChAd:TB vaccine against *M.tb* (H_37_Rv) persisters in the BALB/c model. **A** Experimental schema, pertaining to panel **B**. **B** Lung mycobacterial burden (Log_10_ colony-forming unit (CFU)) 4 weeks post-ABx cessation. **C** Diagram depicting generation of *M*.tb culture filtrates for assessment of mycobacterial persisters in lung homogenates. **D** Left: Most probable number (MPN) estimates (Log_10_) to assess actively replicating bacilli (conventional media, black bars) and persisters (resuscitation media, blue bars). Right: Resuscitation Index, as calculated by a ratio of persisters-to-actively replicating bacilli. **E** Experimental schema, pertaining to panel **F**. **F** Left: Most Probable Number (MPN) estimates to assess actively replicating bacilli (conventional media, black bars) and persisters (resuscitation media, blue bars). Right: Resuscitation Index, as calculated by a ratio of persisters-to-actively replicating bacilli. Data presented in (**B**, **D**, **F**) represent mean ± SEM of *n* = 3 mice/group. Data are representative of one independent experiment. Statistical analysis for (**B**, **D**, **F**) was performed using a nonparametric Kruskal–Wallis with Dunn’s multiple comparison test.

Thus far, we have shown that boosting BCG-primed hosts with i.n. Tri:ChAd:TB provides robust protection against pulmonary TB in comparison to both its monovalent and bivalent counterparts (Fig. 3G). The latter observation strongly suggests that the inclusion of RpfB significantly enhances the efficacy of Tri:ChAd:TB. Given that RpfB is expressed during mycobacterial dormancy and also plays a role during resuscitation, we next investigated whether Tri:ChAd:TB immunization could hinder the development of persisters and thus prevent this immune-evasive strategy of *M.tb*. To address this, we adapted an antibiotic therapy model of pulmonary TB to trigger the development of persisters in vaccinated animals. To this end, 4-week BCG-primed animals were i.n. boosted with either Mono., Biv., or Tri:ChAd:TB. All groups of mice were infected with virulent *M.tb* (H_37_Rv) via the respiratory mucosal route at 4 weeks post-booster immunization. As control, some mice were s.c. immunized with BCG for 8 weeks and infected with *M.tb*. Four weeks after infection all animals were treated via drinking water with triple antibiotic therapy for 2 weeks. Animals were sacrificed at 4 weeks after cessation of antibiotics and lungs were collected for *M.tb* enumeration by conventional solid agar CFU assay (Fig. 4A). In agreement with Fig. 3G boosting with Mono., Biv., and Tri:ChAd:TB markedly reduced bacterial burden in the lung compared to control animals (BCG). Importantly, Tri:ChAd:TB booster immunization rendered the best level of reduction (~3log_10_) in bacterial burden (Fig. 4B).

In the same experimental setup, we also performed limiting dilution, most probable number (MPN) assays which are based on bacterial growth in liquid media. As detailed above^50,64–67^, to quantify the relative burden of both actively replicating and dormant (persister) mycobacteria, we compared the MPN estimates using either liquid media alone (Conventional media), or liquid media supplemented with sterilized *M.tb* culture filtrates (Resuscitation media). Lung homogenates were cultured in resuscitation media to resuscitate persisters into an actively replicating state, allowing for enumeration of both actively replicating bacilli and persisters. Another aliquot of the same lung sample was cultured in conventional media as a control which allows enumeration of only actively replicating bacilli (Fig. 4C). By taking the ratio of these absolute MPNs, we generated a resuscitation index (RI) which provides a numeric representation of the magnitude persisters-to-actively replicating mycobacteria.

Surprisingly, we observed higher MPN counts in samples from BCG-vaccinated animals cultured in Resuscitation media relative to MPN counts from culturing the same samples in Conventional media (1.0log_10_, RI = 7) (Fig. 4D). Such increases were also seen in mono:ChAd:TB-boosted animals (1.10log_10_, RI = 18), and to a lesser extent in Biv:ChAd:TB-boosted animals (0.70log_10_, RI = 5), collectively suggesting the presence of an extensive population of persisters (Fig. 4D). Remarkably, Tri:ChAd:TB-boosted animals showed no differences between culture conditions (0.07_log_10, RI = 1), strongly suggesting that immunization with Tri:ChAd:TB reduced and/or limited the establishment of persisters (Fig. 4D). As detailed above, given that the only difference between our bivalent and trivalent ChAd:TB vectors is the expression of *rpfB*, our data suggests that inclusion of *rpfB* (and therefore RpfB-specific immunity) in Tri:ChAd:TB is capable of hindering persisters.

### Intranasal therapeutic immunization with a multivalent ChAd:TB vaccine significantly reduces nonculturable, persistent *M.tb* bacilli

Immunotherapy adjunct to antibiotic therapy has the potential to significantly accelerate disease control and shorten the duration of conventional antibiotic treatments alone^68,69^. However, the relative impact of adjunct immunotherapy, particularly with therapeutic respiratory mucosal immunization strategies on non-replicating persisters remains to be addressed. Given the superior ability of i.n. Tri:ChAd:TB to prophylactically hinder/reduce the development of non-replicating persisters (Fig. 4D), we next sought to understand whether therapeutic immunization with this vaccine accelerates mycobacterial clearance and reduce the establishment of persisters. To address this, we adapted a vaccine immunotherapy model as described previously^39^. Briefly, 4 weeks after respiratory mucosal *M.tb* (H_37_Rv) infection, mice were treated via drinking water with a triple antibiotic cocktail for 4 weeks. Groups of mice were subsequently i.n. immunized either with Tri:ChAd:TB (Tri.) or its monovalent counterpart (Mono.) at 4 weeks post-initiation of antibiotic therapy. As a control, a group of mice was left unvaccinated. All groups of mice were sacrificed at 4 weeks post-vaccine immunotherapy (Fig. 4E). Bacterial burden in the lung was enumerated using a limiting dilution MPN assay, as described above (Fig. 4C). Immunotherapy with Mono:ChAd:TB did not significantly reduce actively replicating *M.tb* bacilli as measured by MPN assay (Conventional media), whereas Tri:ChAd:TB trended toward reduced actively replicating *M.tb* bacilli in the lung compared to unvaccinated controls (Fig. 4F). In line with these observations, non-replicating persister counts (Resuscitation media) also trended towards being lower in Tri:ChAd:TB-vaccinated hosts compared to unvaccinated controls. Interestingly, immunotherapy with Mono:ChAd:TB promoted the development of persisters as indicated by significantly increased persister counts and higher RI value compared to unvaccinated counterparts. Together, these data suggest that selection of immunotherapy using viral-vectored vaccines adjunct to antibiotic therapy should be done with caution.

### Intranasal immunization with a multivalent ChAd:TB vaccine in parenteral BCG-primed hosts provides enhanced protection against pulmonary infection with *M.tb* in a susceptible murine model

**Fig. 5:**
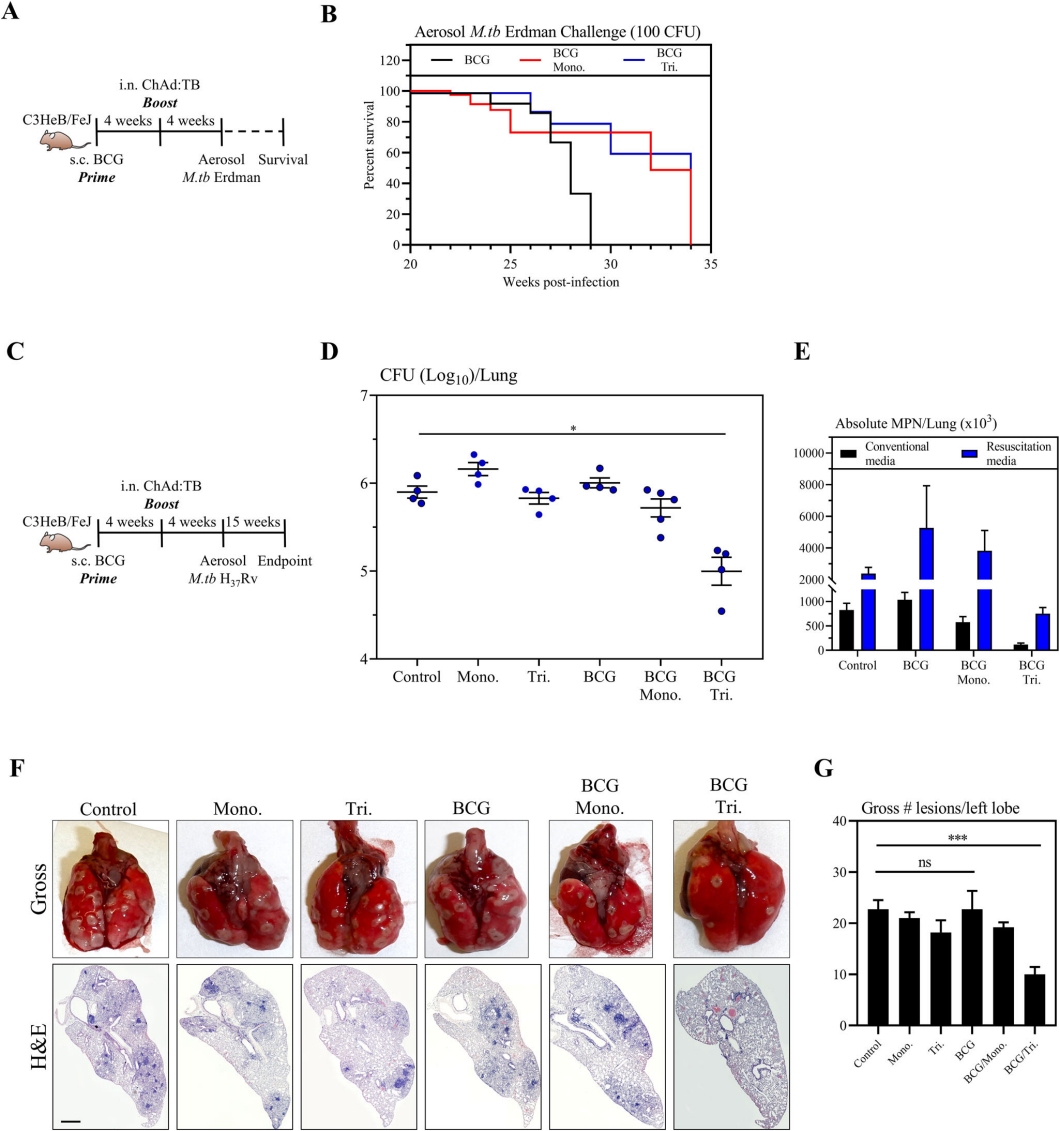
Protective efficacy of a multivalent ChAd:TB vaccine against *M.tb* in the C3HeB/FeJ model. **A** Experimental schema, pertaining to panel **B**. **B** Survival curve following aerosol challenge with *M.tb* (Erdman). **C** Experimental schema, pertaining to panels **D**–**G**. **D** Lung mycobacterial burden (Log_10_ colony-forming unit (CFU)) 15 weeks post-*M.tb* challenge via aerosol. **E** Most Probable Number (MPN) estimates to assess actively replicating bacilli (conventional media, black bars) and persisters (resuscitation media, blue bars). **F** Top: Representative gross lung pathological images 15 weeks post-*M.tb* challenge. Bottom: Representative lung histopathological images of H&E staining 15 weeks post-*M.tb* challenge. Scale bars represent 200 µm. **G** Bar graph depicting total number of gross lesions on the left lungs. Data presented in (**D**, **E**, **G**) represent mean ± SEM of *n* = 4–5 mice/group. Data are representative of one independent experiment. Statistical analysis for (**D**, **G**) was performed using a nonparametric Kruskal–Wallis with Dunn’s multiple comparison test. *P = 0.05, ***P = 0.001.

Our data thus far indicate that our multivalent ChAd:TB vaccine expressing Ag85A, TB10.4, and RpfB robustly protects against both actively replicating and persistent mycobacteria. Importantly, by directly comparing its protective efficacy with a bivalent-vectored ChAd:TB vaccine expressing only Ag85A and TB10.4, our data strongly suggests that vaccine-mediated protection is further enhanced through the inclusion of RpfB into our trivalent vaccine design. However, our observations thus far have been limited to the relatively resistant BALB/c model of tuberculosis infected with a laboratory virulent strain of *M.tb* (H_37_Rv). This model is limited in mimicking characteristics of pulmonary TB seen in humans. Thus, we next validated the protective efficacy of our multivalent ChAd:TB vaccine in the more susceptible C3HeB/FeJ mouse model (hereafter referred to as FeJ mice), which shows numerous hallmarks of human TB disease^70–73^. To investigate the protective efficacy of our multivalent ChAd:TB vaccine in this stringent model, s.c. BCG-primed FeJ mice were i.n boosted with Tri:ChAd:TB or its monovalent counterpart and at 4 weeks post boost were aerosol challenged with virulent *M.tb* (Erdman) (Fig. 5A). As a control, some mice were BCG-primed for 8 weeks and infected. Improved survival was observed in boosted animals (BCG/Mono. and BCG/Tri.) over the control animals (BCG) (Fig. 5B). Although survival rates did not differ significantly between mice boosted with either Mono:ChAd:TB and Tri:ChAd:TB, Tri:ChAd:TB-boosted animals tended to succumb to infection slightly later than those boosted with its monovalent counterpart.

We next examined the capacity of our multivalent ChAd:TB vaccine in controlling pulmonary bacterial burden and limiting pathology in this stringent model. Naive mice or 4-week s.c. BCG-primed mice were i.n. immunized with either Tri:ChAd:TB or its monovalent counterpart. As controls, a group of mice was left unvaccinated (control) or s.c. immunized with BCG for 8 weeks. All animals were aerosol-infected with *M.tb* (H_37_Rv). Mice were sacrificed 15 weeks post infection (Fig. 5C) and bacterial burden was quantified by conventional sold agar CFU assay (Fig. 5D). Compared to control, lung bacterial burden was significantly reduced only in s.c. BCG-primed animals that were boosted with Tri:ChAd:TB vaccine (~tenfold reduction) (Fig. 5D). Given these observations, we next assessed protection against persistent *M.tb* bacilli in BCG-primed, Tri:ChAd:TB-boosted animals, in comparison to relevant control groups (Control, BCG, and BCG Mono:ChAd:TB) (Fig. 5E). Although no statistically significant differences in the counts of replicating and non-replicating persistent *M.tb* bacilli was observed between groups by MPN assay, compared to control, Tri:ChAd:TB vaccine-boosted animals harbored the lowest numbers of both replicating and non-replicating *M.tb* bacilli among all groups (Fig. 5E). Importantly, gross pathological indices reflected by arbitrary scores based on number of lung nodules further indicated significant reduction in lung injury in animals that received Tri:ChAd:TB as a BCG-boost immunization (gross lung images and Fig. 5G). Similarly, significant reduction in granulomatous lesions were observed in the lung of BCG-primed Tri:ChAd:TB-boosted animals (Fig. 5G).

Taken together, these data indicate that even in a stringent model of pulmonary TB, Tri:ChAd:TB, but not the monovalent counterpart, as a BCG booster, provides robust protection by controlling bacterial burden and associated lung immunopathology.

### Intranasal immunization with a multivalent ChAd:TB vaccine provides enhanced protection against pulmonary *M.tb* infection in a humanized mouse model

**Fig. 6:**
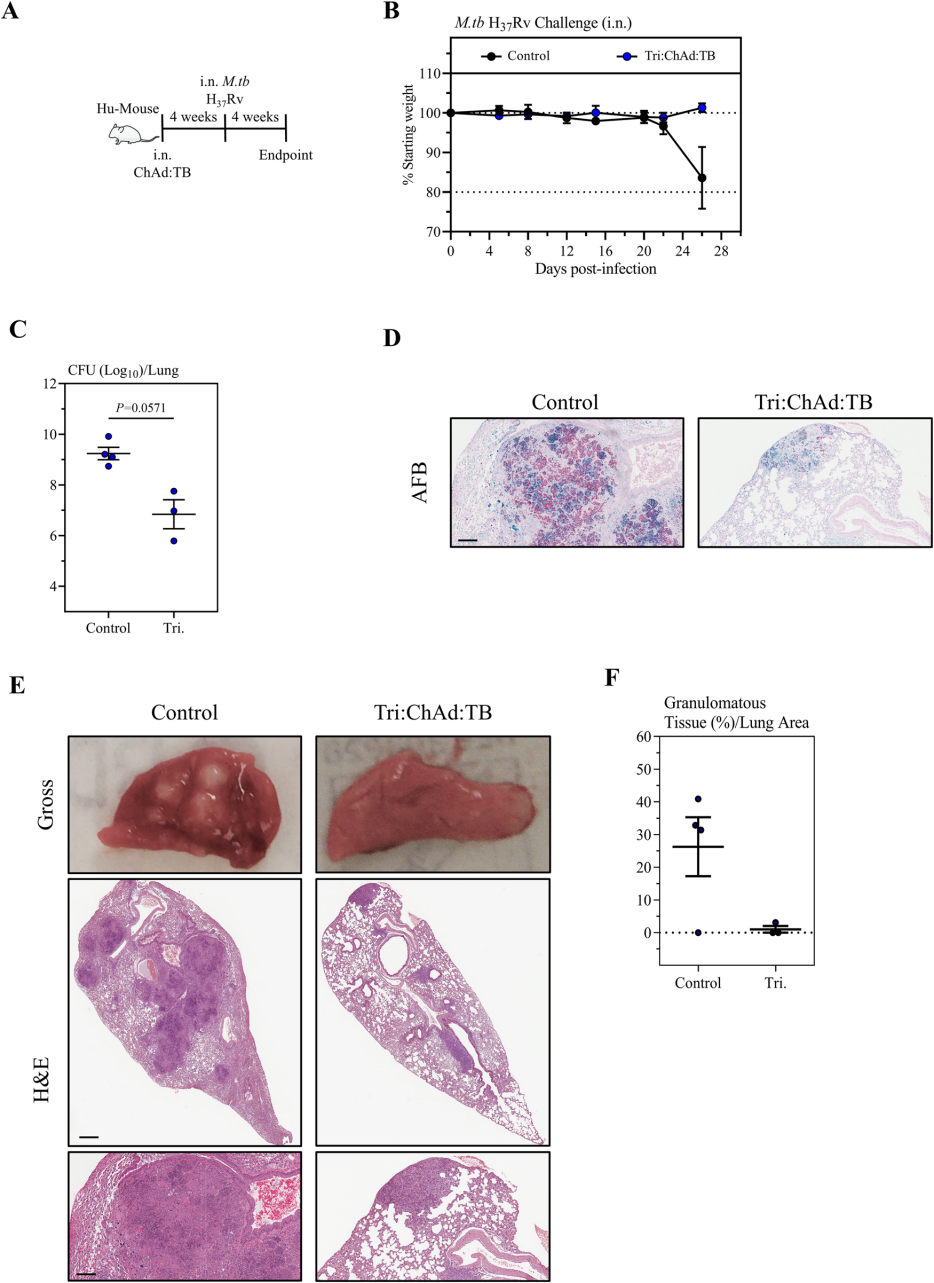
Protective efficacy of a multivalent ChAd:TB vaccine against *M.tb* (H_37_Rv) in the humanized mouse (Hu-mouse) model. **A** Experimental schema. **B** Body weight change curve following respiratory mucosal challenge with *M.tb* (H_37_Rv). **C** Lung mycobacterial burden (Log_10_ colony-forming unit (CFU)) 4 weeks post-*M.tb* challenge. **D** Representative lung histopathological images of acid-fast Bacilli (AFB) staining 4 weeks post-*M.tb* challenge. Scale bars represent 100 µm. **E** Top: Representative gross lung pathological images Scale bars represent 200 µm. Bottom: Representative lung histopathological images of H&E staining. Scale bars represent 100 µm. **F** Scatter plots depicting percentage areas of granulomatous lesions in the lungs. Data presented in (**B**, **C**, **F**) represent mean ± SEM of *n* = 3–4 mice/group. Data are representative of one independent experiment. Statistical analysis for (**C**) was performed using a nonparametric Mann–Whitney *T* tests.

Preclinical murine models are indispensable in the development and evaluation of novel TB vaccination strategies. However, clinical translatability from these models is ultimately limited given that *M.tb* is solely a human pathogen^74^. Although non-human primates help further bridge preclinical findings, their utility is limited by logistical and ethical considerations. As such, humanized mice (Hu-mouse), that harbor a human immune system have emerged as attractive models to address such limitations^75–77^. We have previously shown that i.n. immunization in a Hu-mouse model with a well-characterized monovalent adenoviral-vectored vaccine induces human T-cell responses which are directly able to restrict human-like pulmonary TB disease^78^. Given the protective efficacy observed with our multivalent ChAd:TB vaccine in two conventional mouse models, we next sought to evaluate this vaccine in the Hu-mouse model as a means to assess its potential clinical relevance. Humanized mice were generated as previously described^78^. Briefly, irradiated newborn *NOD-Rag1*^*tm1Mom*^*Il2rg*^*tm1Wjl*^ (NRG) mice were reconstituted with CD34^+^ hematopoietic stem cells enriched from human cord blood. Engraftment was confirmed by flow cytometry at 12 weeks post-reconstitution (Table 1). Given logistical constrictions in generating Hu-mice, we selected to only proceed with i.n. Tri:ChAd:TB immunization in BCG-naive hosts as a means to directly assess the protective efficacy of our vaccine in this stringent mouse model. We randomized animals to either remain unvaccinated or be i.n. immunized with Tri:ChAd:TB. Animals were challenged with *M.tb* (H_37_Rv) via the respiratory mucosal route at 4 weeks post immunization, and were monitored for weight loss as an indices of TB disease (Fig. 6A). In the same experiment, animals were sacrificed at pre-defined time, 4 weeks post infection, to quantify lung mycobacterial burden and evaluate lung injury (Fig. 6A). Given the highly susceptible nature of Hu-mice to TB, we observed considerable weight loss among unvaccinated animals, averaging ~20%. In contrast, at this timepoint all i.n. Tri:ChAd:TB-immunized animals did not lose weight (Fig. 6B). In congruence with these clinical observations, we observed a significant reduction in mycobacterial burden in the lung as determined by CFU assay in Tri:ChAd:TB-immunized mice (2.40log_10_ reduction compared to control) (Fig. 6C). Enhanced bacterial control in immunized mice were further supported by markedly reduced densities of acid-fast bacilli (AFB) in the microscopic lung sections (Fig. 6D). In addition, lungs from immunized animals showed remarkably reduced gross pathological changes (Fig. 6D) and microscopic granulomatous lesions (Fig. 6E). Collectively, the above data indicate that i.n. immunization with our multivalent ChAd:TB vaccine can provide robust protection against TB in a clinically translatable and highly susceptible humanized mouse model.

**Table 1:** Engraftment of human immune cells in humanized mice.

Group	% HuCD45	% HuCD3	% HuCD4	% HuCD8
Control	25.50	55.70	54.90	32.60
Tri:ChAd:TB	15.80	52.80	46.50	37.40

The blood from humanized mice used in control and Tri:ChAd:TB-vaccinated groups was examined by flow cytometry for verification of human immune engraftment.

## Discussion

The success of *Mycobacterium tuberculosis* as a human pathogen in part stems from its multistage life cycle, whereby it can readily adapt to unfavorable environmental and host immune pressures^79^. Metabolic and concomitant antigenic changes that occur when transitioning from an actively replicating, to a dormant, persistent state has profound negative effects on the efficacy of front-line TB therapeutics^80^. Persisters are remarkably tolerant to the bactericidal mechanisms of many TB antibiotics, thereby representing a major hurdle contributing to treatment duration, regimen default, and disease relapse^64^. In fact, it has recently been suggested that most active TB cases may arise from latent disease, due to *Mycobacterial* resuscitation from a state of dormancy^81^. Antigenic changes due to host pressures also allow *M.tb* to evade host adaptive immune responses established during natural infection^23,79,82^. These observations are mirrored when the bacilli encounter vaccine-mediated immune responses, which may be rendered ineffective as a consequence of a shifting antigenic profile. As such, we postulate that the lack of effectiveness of monovalent recombinant vaccines which express a singular antigen expressed solely during acute infection may in part be due to changes in antigen expression as *M.tb* transitions to- and from a persistent state. In this regard, there has been increased interest in the development of vaccine strategies that include antigens from multiple stages of the *M.tb* life cycle^26,32,33,83,84^. In this study, we have taken these factors into consideration and have developed a next-generation multivalent chimpanzee adenoviral-vectored vaccine (Tri:ChAd:TB) encoding multiple antigens (*Ag85A, TB10.4*, and *rpfB*) that have been shown to be expressed across the multiple stages of the *M.tb* life cycle (Fig. 1)^42^.

We have shown that i.n. immunization with our multivalent ChAd:TB vaccine induces long-lasting tissue-resident memory CD4^+^ and CD8^+^ T cells against Ag85A, TB10.4, and RpfB within the airways which are further boosted quantitively and qualitatively in s.c. BCG-primed hosts. Importantly, i.n. immunization with Tri:ChAd:TB either as a single i.n. dose or as a BCG booster provides remarkable prophylactic protection against both mycobacterial infection and lung immunopathology by two virulent *M.tb* strains in three separate murine models, including a clinically relevant and highly stringent humanized mouse model (Figs. 2–6). In line with these observations, we further show that therapeutic vaccination with Tri:ChAd:TB adjunct to conventional TB antibiotic therapy, can accelerate TB control. Furthermore, for the first time we provide evidence as pertaining to persistent *M.tb* subpopulations in a vaccine model that by incorporating antigens such as RpfB into vaccine design we can further limit/reduce such difficult-to-eliminate subpopulations (Figs. 4 and 5).

Our study further strengthens the importance of the immunization route in the development of novel TB vaccine strategies^4^. The vaccination route dictates the quality and localization of the resultant immune responses. As such, the poor efficacy of parenterally delivered vaccines such as BCG or MVA85A against pulmonary TB may stem from the fact that vaccine-derived immunity is confined to the periphery^61^. Our study highlights not only that i.n. immunization with a multivalent TB vaccine establishes multifunctional, long-lasting tissue-resident memory T cells within the airway and lung tissues, critical to durable protection, but also that BCG-specific T cells restricted to the periphery may be boosted and subsequently drawn into the airways, potentially further contributing to anti-TB immunity (Fig. 2). Indeed, we show readily detectable TB10.4- and RpfB-specific T-cell responses drawn to the airway following i.n. boosting with Mono:ChAd:TB, suggesting that these T-cell responses were primed by BCG and subsequently pulled into the airways by respiratory mucosal boosting (Fig. 2D, E). It is noteworthy that although our study assessed BCG-boosting responses only at 2 weeks post immunization, we have previously shown that such anamnestic responses are maintained much longer, upwards of 4 weeks post boost^40^.

Our findings also indicate the relevance of the breadth of immune responses induced by a TB vaccine. By expressing Ag85A, TB10.4, and RpfB, animals immunized with Tri:ChAd:TB had significantly reduced pulmonary mycobacterial burden which was associated with markedly reduced lung immunopathology in comparison to animals immunized with the vaccines that lacked the antigenic breadth or did not span the spectrum of the *M.tb* life cycle (Fig. 3). Specifically, we have shown that in contrast to our monovalent or bivalent vaccine which express either Ag85A alone, or Ag85A and TB10.4, respectively, that inclusion of all three antigens provides greater protection against not only actively replicating bacilli but also dormant, persistent subpopulations (Fig. 4). In line with these observations, we have also shown that therapeutic immunization with Tri:ChAd:TB is effective against persisters emerged following cessation of antibiotic therapy. Such therapeutic protection is in stark contrast to the failure of control of the persisters by the monovalent vaccine despite its induction of comparable levels of Ag85A-specific T-cell immunity (Fig. 4E, F). These observations suggest that vaccine strategies solely targeting the early secreted antigens of *M.tb* might not be an effective prophylactic/immunotherapeutic approach.

There are multiple multistage TB vaccine candidates currently under investigation, including those based on recombinant BCG strains, which have shown some degree of immunity against active and latent TB^26,32,81,84^. However, such strategies are not fully suitable for respiratory mucosal administration. Given the data presented here and in other previously published studies, next-generation TB vaccination strategies should take into consideration respiratory mucosal delivery in their design. Thus, viral-vectored multivalent TB vaccines which are amenable for respiratory mucosal delivery, such as the one evaluated in the current study and others currently under investigation, may be more effective in conferring anti-TB immunity at the portal of infection. Although induction of mucosal immunity has been shown to be critical in protection against TB, not all vectors and/or antigens may provide such enhanced protection, highlighting the complexities in TB vaccine design, and the importance of testing novel vaccine candidates in a variety of preclinical models^85,86^.

Our current study has for the first time examined the heterogenous populations (replicating and non-replicating persistent) of *M.tb* bacilli in the lung as a marker of protective efficacy of a multivalent vaccine in a variety of murine models including the highly susceptible FeJ mouse and humanized mouse models. Although the presence of *M.tb* persisters has been demonstrated in sputum samples of TB patients and following antibiotic therapy^65^, the analysis in the lung of preclinically vaccinated animals has never been carried out before. Here, we have shown that a heterogeneous population of *M.tb* bacilli is present in the lungs of both unvaccinated and vaccinated hosts and have also demonstrated that by including the dormancy/resuscitation-associated antigen RpfB into our vaccine design, we can limit/reduce persisters (Figs. 4 and 5E). Rpf proteins have been shown to be immunogenic in humans and significant T-cell responses were demonstrated in LTBI individuals against Rpf which had linked to strengthened immune surveillance in LTBI individuals and prevention of reactivation^28,50^. The methodology utilized in our study to quantify mycobacterial persisters must in part be interpreted with caution. Although previous studies have use similar methods to measure this occult population, there remains the possibility that the culture filtrate supernatants used in resuscitation may also promote expansion of actively replicating bacilli as well. As such, although our results presented in Figs. 4 and 5 clearly indicate that our trivalent ChAd:TB vaccine is capable of reducing persisters, it cannot be ruled out that the MPN values may include both actively replicating and persister subpopulations. The inhibitory effect and mechanisms of this vaccine on the persisters warrants a further follow-up study. Apart from the proper assessment of heterogenous *M.tb* populations in vaccine evaluation studies, the validity of the experimental animal models of pulmonary tuberculosis is also critical. Genetic heterogeneity of mice and the dose and virulence of *M.tb* used for infection can all influence the protective outcomes and clinical relevance in vaccine evaluation studies. Using the most stringent FeJ murine model of tuberculosis, which develops necrotizing granuloma more consistent with human tuberculosis disease, we demonstrated that boosting with a multivalent viral-vectored vaccine, Tri:ChAd:TB can prolong the survival of *M.tb* infected hosts, which was associated with markedly reduced replicating and non-replicating dormant *M.tb* bacilli and necrotizing granuloma in the lung (Fig. 5). Tri:ChAd:TB vaccine conferred protective efficacy in FeJ mice following both Erdman and H_37_Rv aerosol *M.tb* challenge. In this study, BCG or viral-vectored vaccine as a stand-alone vaccine failed to protect FeJ mice, different from its protective effects in BALB/c mice. This is likely related to the longer time post infection (15 weeks) chosen to study the protective efficacy as compared to other studies which mostly studied protective efficacy at 4 weeks post-vaccination^87^. In this regard, intranasal Tri:ChAd:TB immunization in BCG-primed hosts strongly boosted CD4^+^ T-cell responses to TB10.4 and RpfB (Fig. 2E), which could have contributed to the markedly enhanced protection. Furthermore, our observation that intranasal Tri:ChAd:TB immunization also significantly protected the highly susceptible humanized animals from pulmonary TB disease further highlights the potential of this multivalent TB vaccine strategy. This is in line with our previous study where prophylactic intranasal immunization with a human adenovirus-vectored monovalent TB vaccine demonstrated a level of protection in humanized mice. Such protection was linked to antigen-specific T cells induced by the vaccine^78^.

In summary, we show that the multivalent viral-vectored next-generation tuberculosis vaccine delivered via the respiratory mucosal route remarkably enhances protection against pulmonary tuberculosis in three separate murine models including a highly clinically relevant humanized mouse model. Of importance, we provide evidence that this vaccine is able to expand protective efficacy by controlling both replicating and non-replicating dormant *M.tb* bacilli in the lung. We further show that this vaccine strategy could accelerate TB control adjunct to TB antibiotic therapy.

## Methods

### Animals for in vivo studies

Female BALB/c mice (6–8 weeks old) were purchased from Charles River (Wilmington, MA, USA). Female C3HeB/FeJ mice (6–8 weeks old) were purchased from The Jackson Laboratory (Bar Harbor, ME, USA). All experimental mice were housed within either the level 2 or level 3 containment facility at McMaster University.

### Ethical statement

All experiments were carried out according to the animal utilization protocol (AUP# 210822) approved by the Animal Research and Ethics Board of McMaster University. They were housed under the conditions described above with ad libitum access to food and water, 12 h light cycle, 50–60% humidity, and at 20–25 °C room temperature.

### Development and immune screening of humanized mice

NOD-Rag1null IL2rgnull (NRG) mice were obtained from Jackson Laboratories (Bar Harbor, Maine, USA). NRG pups (24–72 h old) were irradiated twice with 3 cGy 3 h apart and then engrafted intrahepatically with 10^5^–10^6^ CD34^+^ hematopoietic stem cells (HSC) isolated from human umbilical cord blood. At 12 weeks post engraftment, blood was collected to quantify human immune cell reconstitution using flow cytometry. Erythrocytes were lysed using an ACK lysis buffer and the remaining cells were treated with both anti-human Fc Receptor Binding Inhibitor and anti-mouse CD16/CD32 antibodies (eBiosciences). Cells were then stained with an antibody cocktail (mCD45-AlexaFluor 700 (1:50), hCD45-Pacific Blue (1:20), hCD3e-Qdot 605 (1:100), hCD4-PerCP-Cy5.5 (1:20), hCD8a-PE-Cy7 (1:20)), followed by fixable viability dye (APC-eFluor 780; eBiosciences). Samples were run on the Cytoflex LX flow cytometer equipped with a flow rate calibrator and analyzed using FlowJo software version 10 (Tree Star, Ashland, OR, USA). Mice with at least 10% or 50,000 per mL hCD45+ leukocytes in the blood were selected for subsequent experiments.

### Molecular construction and validation of a multivalent chimpanzee adenovirus vaccine

A replication-deficient chimpanzee serotype 68 adenovirus was constructed to express three *M.tb* antigens-Antigen 85A, TB10.4, and RpfB. Each antigen is separated by linkers composed of four glycine residues (Tri:ChAd:TB), using previously reported technology^40^. The transgene cassette was cloned to express the murine cytomegalovirus promoter (MCMV), and a tissue plasminogen activator peptide signal. Apart from the expressed antigens, this trivalent vector is molecularly identical to the monovalent vector (Mono:ChAd:TB) expressing Ag85A alone. Briefly, a pShuttle plasmid was engineered to express the transgene cassette and amplified in DH5α *E.coli* (ThermoFisher, Waltham, MA, USA). The entire transgene cassette was excised and subcloned into the DNA clone pAdCh68 (ΔE1/E3) by I-Ceu1/PI-Sce1 digest and subsequent in-gel ligation. Trivalent AdCh68 was subsequently packaged and propagated in HEK 293 cells and purified by cesium chloride centrifugation. Transgene expression was characterized by western blot. A549 cells were infected with Tri:ChAd:TB at an MOI of 10 and protein was isolated 24 h post infection. Protein lysates were prepared with RIPA buffer containing protease inhibitors (ThermoFisher, Waltham, MA, USA). The lysates were quantified with a BCA kit (ThermoFisher Scientific Waltham, MA, USA) and 20 μg of each lysate was boiled at 98 °C with 1× sample buffer (6.35% v/v 1 M Tris, pH 6.8, 46.5% v/v 10× SDS, 20% v/v glycerol, and 5% v/v β-mercaptoethanol; (MilliporeSigma, Etobicoke, ON, Canada) for 10 min. Samples were run on a 4–12% SDS-PAGE gel (ThermoFisher Scientific Waltham, MA, USA) for 1.5 h at 100 V and transferred to nitrocellulose membrane (VWR, Mississauga, ON, Canada) using wet-transfer at 125 mA for 1.5 h. The membrane was blocked with a 5% skim milk in a tris-buffered saline with 0.05% Tween (TBST-T) solution. The membrane was subsequently probed with a mAb against TB10.4 (kindly provided by Dr. Jes Dietrich) overnight at 4 °C. The membrane was subsequently washed with TBST before being probed with an anti-mouse IRDye secondary antibodies (LI-COR, Lincoln, NE, USA) diluted in 5% skim milk. The membrane was developed using an Odyssey CLx (LI-COR, Lincoln, NE, USA).

### Intranasal immunization with Tri:ChAd vaccines

Immunization was done by intranasal instillation of 1 × 10^7^ PFU of ChAd:TB vaccines in a total volume of 25 μL of sterile phosphate-buffered saline (PBS). In select experiments, BCG (Pasteur) immunization was performed subcutaneously at a dose of 1 × 10^5^ CFU in 100 μL of sterile PBS.

### *M. tuberculosis* infection and antibiotic therapy

Mice were infected either via the respiratory mucosal route (intranasal), or aerosol inhalation utilizing a Glas-Col inhalation exposure system (Glas-Col, LLC, Terre Haute, IN, USA). Intranasal infections were carried out with 1 × 10^4^ colony-forming units (CFU) of *M. tuberculosis* H_37_Rv (ATCC27294) in a total volume of 25 µL of sterile PBS, achieving an infectious inoculum of ~1000 CFU 1 day post infection. Aerosol inhalation infections were carried out with either *M. Tuberculosis* H_37_Rv (ATCC27294) or *Tuberculosis* Erdman (kindly provided by Dr. David Russell), achieving an infectious inoculum of ~10–100 CFU one day post infection. Mice which received antibiotics for therapeutic immunization studies did so in an oral Medidrop (Clear H_2_O, Westbrook, ME, USA) solution ad libitum which included rifampicin (10 mg/kg), isoniazid (25 mg/kg), and pyrazinamide (150 mg/kg). The Medidrop antibiotic solution was replaced weekly, and maintained as per experimental requirements.

### Mononuclear cell isolation

Lungs were cut into small pieces and digested with 150 units of collagenase type 1 (Life Technologies, Grand Island, NY, USA) in RPMI medium at 37 °C with agitation for an hour. Digested lung pieces were then crushed through a 100-µm filter and red blood cells were removed by treatment with an ACK lysis buffer. BAL cells were isolated by centrifugation. Splenic mononuclear cells were isolated by crushing the organ through a 100-µm filter, with red blood cells being removed by treatment with an ACK lysis buffer. Cells were resuspended in RPMI supplemented with 10% FBS, 1% pen-strep, 1% l-glutamine.

### Cell stimulation, intracellular cytokine staining, and flow cytometry

Mononuclear cells were cultured in U-bottom plates at a concentration of 20 million cells per mL. For stimulation, 5 µg/well of either recombinant Ag85A, RpfB, and/or TB10.4 were used. Cells were stimulated for a total of 6 h in the presence of brefeldin A (5 mg/Ml; BD Pharmingen, San Jose, CA, USA). In select experiments, BCG-specific immune responses were assessed by stimulation with crude BCG and *M.tb* culture filtrate at a concentration of 1 µg/mL, in the presence of brefeldin A. Following incubation, cells were washed and blocked with CD16/CD32 FcBlock (1:100, cat# 553142) in 0.5% bovine serum albumin/PBS for 15 min on ice, prior to being stained with the experimental-specific fluorochrome-labeled monoclonal antibodies, according to the manufacturer’s instructions (BD Pharmingen). This included: T-cell panel - CD3-V450 (1:200, cat# 560801), CD8a–PE–Cy7 (1:400, cat# 561097), CD4– APC–Cy7, (1:400, cat# 552051) IFN-γ–APC (1:150, cat# 554413), TNFα-FITC (1:100, cat# 561064), PE-IL-2 (1:100, cat# 554428). Neutrophil and monocycle panel – CD45-APC–Cy7 (1:400, cat# 557659), CD11b-PE-Cy7 (1:400), Ly6C-Biotin (1:200, cat# 557359), Streptavidin-QDot800 (1:500, cat# 10171MP) and Ly6G-BV605 (1:500, cat# 563005) (from BD Biosciences, or ThermoFisher Scientific). All flow cytometry data were collected using a Fortessa Cytometer and FACSDiva software (BD Biosciences, San Jose, CA, USA) and analyzed using FlowJo software version 10 (Tree Star, Ashland, OR, USA).

### Measurement of tuberculosis disease outcomes

Lung mycobacterial bacillary load was assessed by plating serially-diluted lung homogenates on Middlebrook 7H10 agar plates supplemented with 10% OADC growth supplement (BD Biosciences, San Jose, CA, USA), 5 µg/mL ampicillin and 50 µg/mL cycloheximide (Sigma-Aldrich, St. Louis, MO, USA). Lung pathology was assessed histologically using lungs embedded in paraffin and assessed following either hematoxylin and eosin (H&E) or Ziehl-Neelson acid-fast staining. Histological samples were visualized by the Zeiss M2 Imager System (Zeiss, Toronto, ON, Canada). Lung lesions and pulmonary granulomatous lesions were determined quantitatively by using Image J software (NIH, http://rsb.info.nih.gov/nih-image/) by measuring the areas of dense inflammatory infiltrates relative to the total lung sample area. For each animal, three independent lung slices were measured prior to being averaged. Quantification was performed in an unbiased fashion by trained individuals blinded to the origins of the samples and groups.

### Mycobacterial resuscitation assay

Lung homogenates from infected animals were cultured in flat bottom plates in either conventional media (7H9 media supplemented with 10% (vol/vol OADC (BD Biosciences) and 0.05% Tween80) or a resuscitation media. Resuscitation media was extracted from culture supernatants isolated from *M.tb* cultures grown in supplemented 7H9 media to the mid-exponential stage (OD_600 nm_ 1.4). Briefly, bacteria were removed by centrifugation prior to being filtered twice through a 0.2-µm filter. Resuscitation media was composed of 50% of conventional media (vol/vol). Media was supplemented with polymyxin B (200 U/mL), carbenicillin (100 µg/mL), trimethoprim (20 µg/mL), and amphotericin B (10 µg/mL). Most probable numbers were calculated as described^88^, using https://www.wiwiss.fu-berlin.de/fachbereich/vwl/iso/ehemalige/professoren/wilrich/MPN_ver6.xls.

### Statistical analysis

Nonparametric Mann–Whitney *t* tests were carried out for comparison between two groups. Nonparametric Kruskal–Wallis with Dunn’s multiple comparison tests were carried out for multiple-group comparison using GraphPad Prism 8 software (Version 8, La Jolla, CA, USA). Results were considered significant for *P* values ≤ 0.05.

### Reporting summary

Further information on research design is available in the Nature Research Reporting Summary linked to this article.

## Supplementary information

Supplemental Information

REPORTING SUMMARY

## Data Availability

All relevant data generated during this study are available upon reasonable request made to correspondence authors.

## Materials availability

All requests for resources and reagents should be directed to and will be fulfilled by the lead contact or correspondence authors. All reagents including antibodies, viruses, and vaccines may be made available on request after the completion of a Materials Transfer Agreement.
