# Supplementary material for: Intranasal multivalent adenoviral-vectored vaccine protects against replicating and dormant M.tb in conventional and humanized mice
Source: NPJ Vaccines. 2023 Feb 23;8:25. doi: 10.1038/s41541-023-00623-z (PMC9948798; doi:10.1038/s41541-023-00623-z)
Supplement: Supplementary file 1 — Supplemental Information [file 41541_2023_623_MOESM1_ESM.pdf]

Supplemental figure 1.

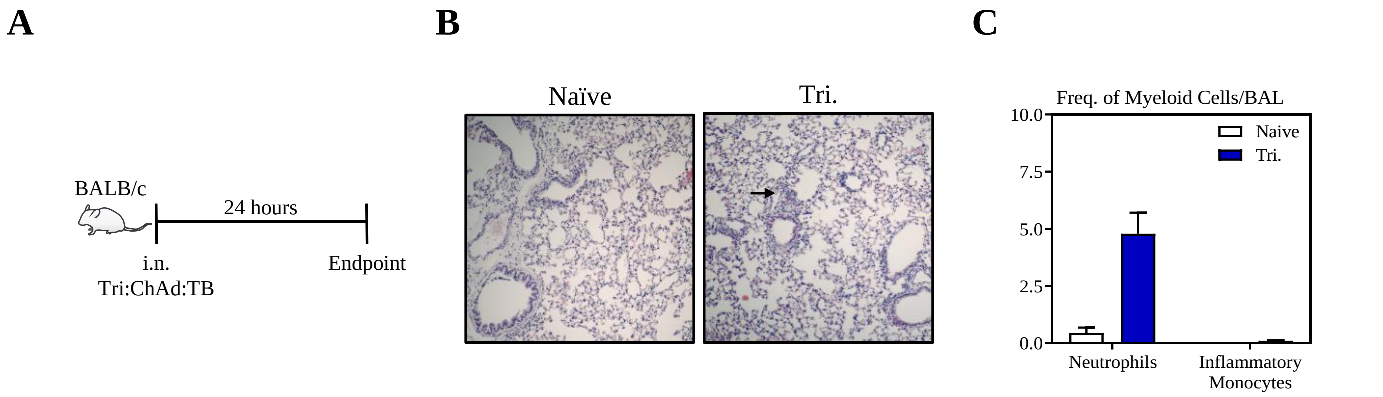

Supplemental Figure 1. Safety profile of a multivalent ChAd:TB vaccine.

A. Experimental Schema.

B. Representative lung H&E images from either naïve animals, or 24 hours post respiratory mucosal vaccination with Tri:ChAd:TB. Black arrows indicate areas of cellular infiltration.

C. Bar graph depicting frequency of myeloid cells in the BAL from naïve and Tri:ChAd:TB-immunized animals.

Supplemental figure 2.

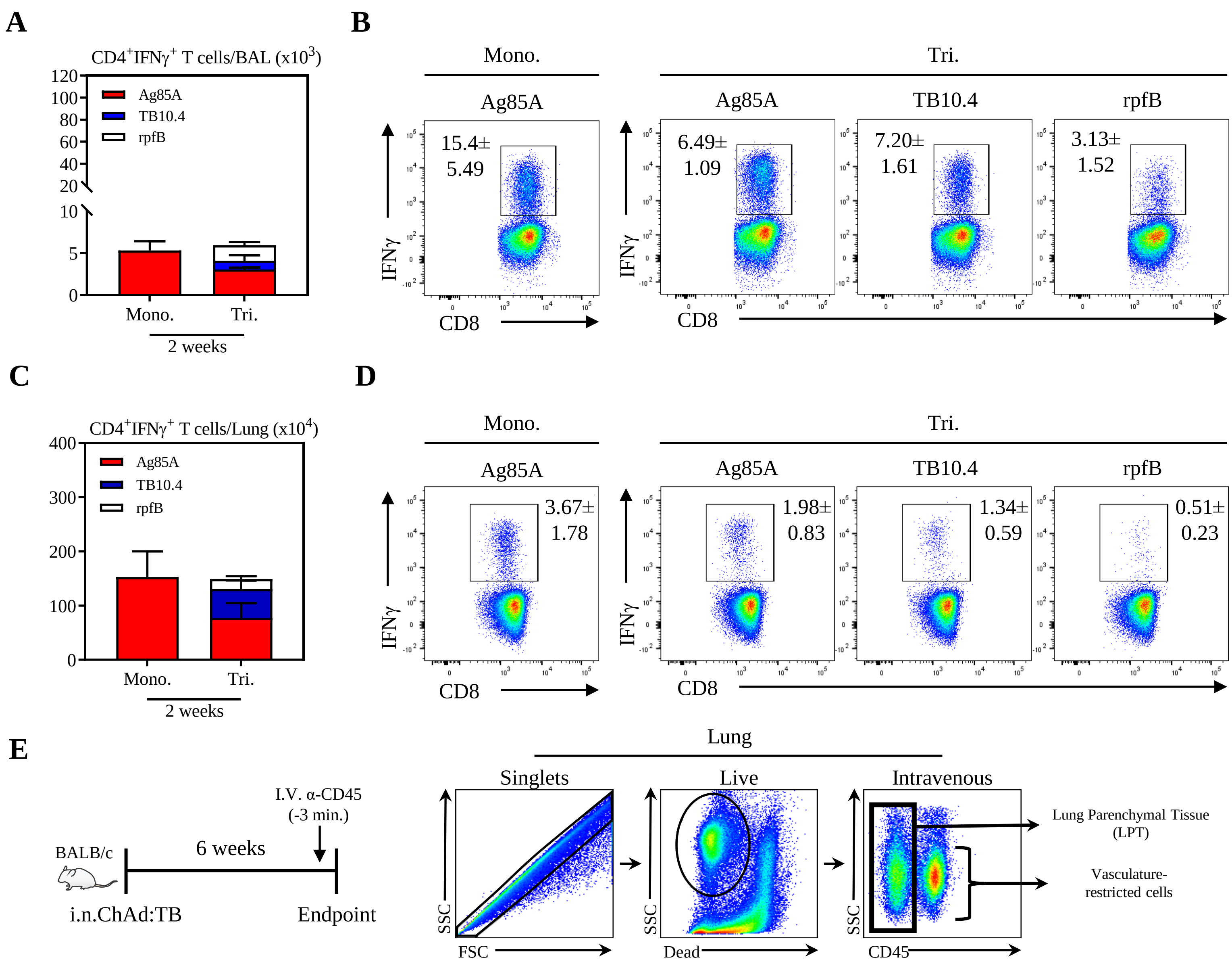

Supplemental Figure 2. Immunogenicity of a multivalent ChAd:TB vaccine.

A. Stacked bar graphs depicting absolute numbers of $CD8^+$ T cell responses in the BAL two weeks post-intranasal (i.n.) vaccination with either Mono:ChAd:TB or Tri:ChAd:TB, as measured by expression of IFNγ following *ex vivo* stimulation with Ag85A (red), TB10.4 (blue), or RpfB (white) whole protein.

B. Representative flow cytometric plots of $IFN\gamma^+CD8^+$ T cells in the BAL two weeks post-i.n. immunization with either Mono:ChAd:TB or Tri:ChAd:TB, following *ex vivo* stimulation with Ag85A, TB10.4, or RpfB whole protein.

C. Stacked bar graphs depicting the absolute number of $CD4^+$ T cell responses in the lung two weeks post-i.n. immunization with either Mono:ChAd:TB or Tri:ChAd:TB, as measured by expression of IFNγ following *ex vivo* stimulation with Ag85A (red), TB10.4 (blue), or RpfB (white) whole protein.

D. Representative flow cytometric plots of $IFN\gamma^+CD4^+$ T cells in the lung two weeks post-i.n. immunization with either Mono:ChAd:TB or Tri:ChAd:TB, following *ex vivo* stimulation with Ag85A, TB10.4, or RpfB whole protein.

E. Left: Experimental schema. Right: Representative flow cytometric plots depicting localization of immune cells in either the lung parenchymal tissue (LPT) or lung vasculature.

Data presented in (A-E) represent mean ± SEM of n=3 mice/group. Data is representative of 1 independent experiment.

Supplemental figure 3.

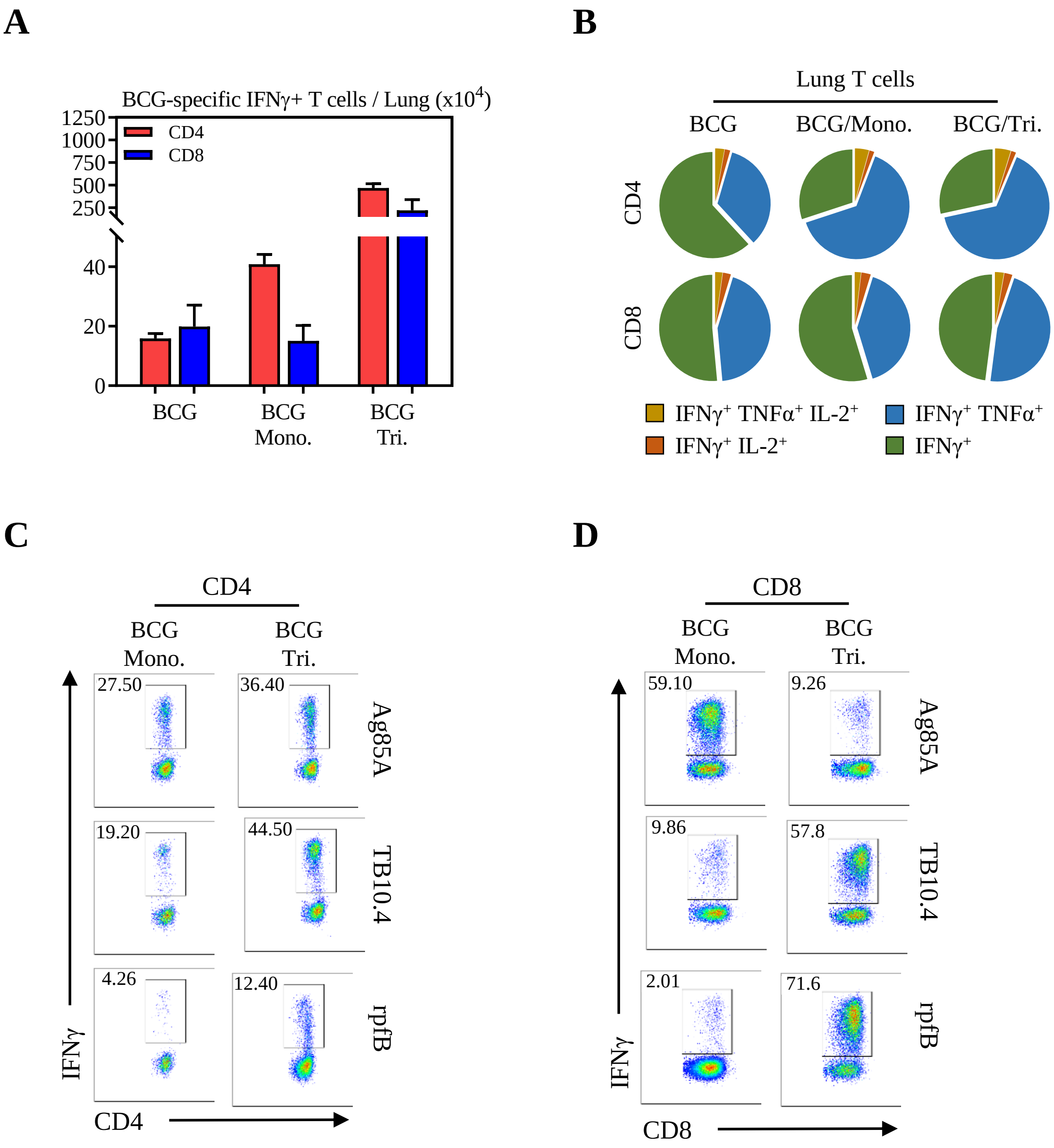

Supplemental figure 3. Immunogenicity of a multivalent ChAd:TB vaccine in BCG-primed animals.

A. Bar graphs depicting absolute numbers of either CD4^{+} (red) or CD8^{+} (blue) T cell responses in the lung, as measured by expression of IFNγ following *ex vivo* stimulation with crude BCG/culture filtrate.

B. Pie charts depicting the functionality (IFNγ, TNF-α, and/or IL-2) of CD8^{+} or CD4^{+} T cells following *ex vivo* stimulation with crude BCG/culture filtrate.

C. Flow cytometric plots of IFNγ^{+}CD4^{+} T cells in the BAL following i.n. immunization with either Mono:ChAd:TB or Tri:ChAd:TB, following *ex vivo* stimulation with Ag85A, TB10.4, or RpfB whole protein.

D. Flow cytometric plots of IFNγ^{+}CD8^{+} T cells in the BAL following i.n. immunization with either Mono:ChAd:TB or Tri:ChAd:TB, following *ex vivo* stimulation with Ag85A, TB10.4, or RpfB whole protein.

Data presented in (A) represent mean ± SEM of n=3 mice/group. Data is representative of 1 independent experiment.

Supplemental figure 4

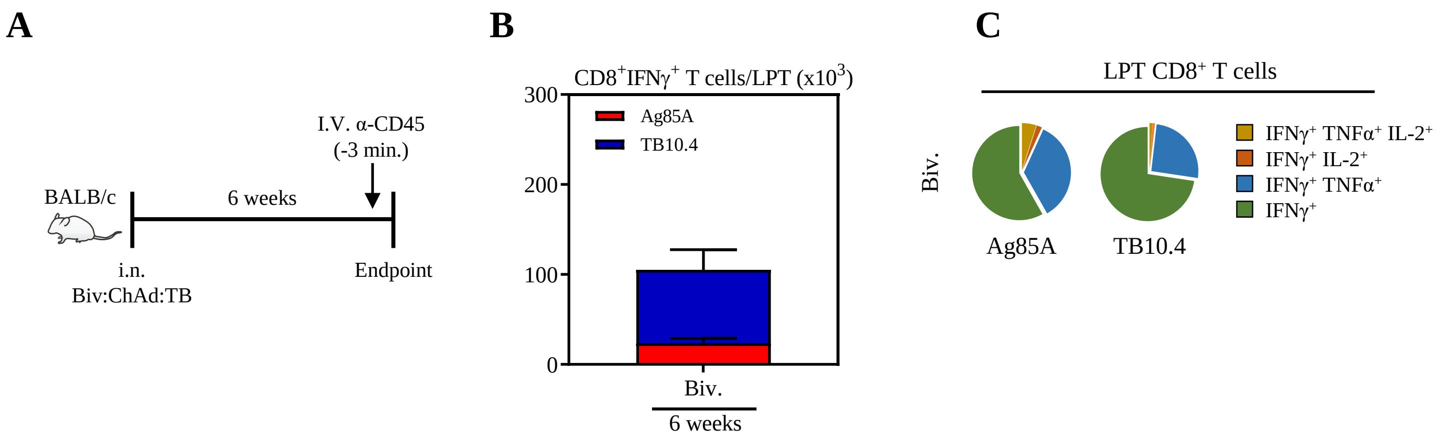

Supplemental Figure 4. Immunogenicity of a bivalent ChAd:TB vaccine.

A. Experimental schema

B. Stacked bar graphs depicting absolute numbers of $CD8^{+}IFN\gamma^{+}$ T cell responses in the LPT 6 weeks post-intranasal (i.n.) vaccination with Biv:ChAd:TB, as measured by expression of IFNγ following *ex vivo* stimulation with Ag85A (red), or TB10.4 (blue).

C. Pie charts depicting the functionality (IFNγ, TNFα, and/or IL-2) of LPT $CD8^{+}$ T cells 6 weeks post-i.n. immunization with Biv:ChAd:TB, following *ex vivo* stimulation with Ag85A, or TB10.4.

Data presented in (A) represent mean ± SEM of n=3 mice/group. Data is representative of 1 independent experiment.

Supplemental figure 5

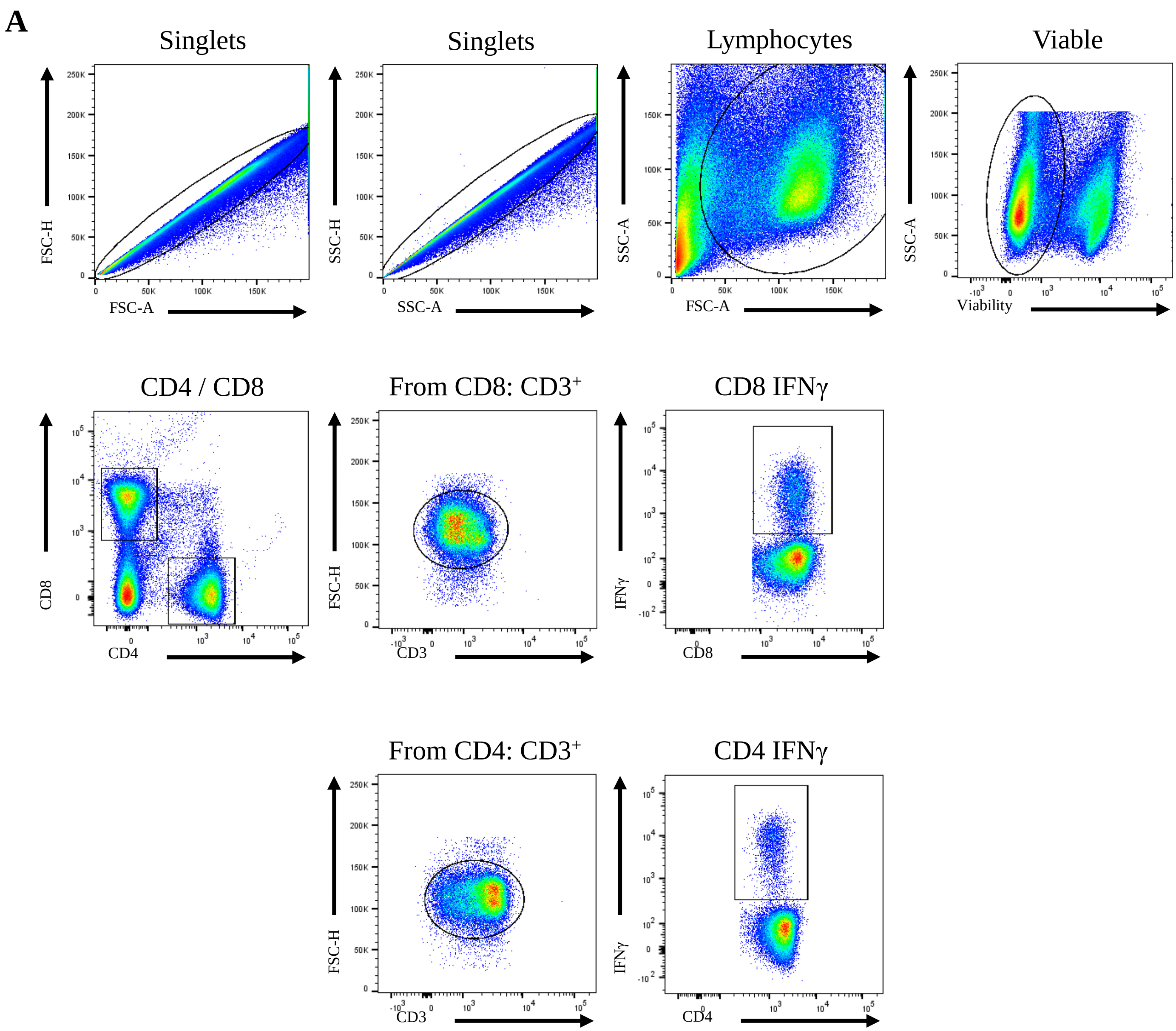

Supplemental Figure 5. T cell gating strategy.

A. Gating strategy for identification of cytokine producing CD4^{+} or CD8^{+} T cells.
