# Supplementary material for: Intranasal multivalent adenoviral-vectored vaccine protects against replicating and dormant M.tb in conventional and humanized mice
Source: NPJ Vaccines. 2023 Feb 23;8:25. doi: 10.1038/s41541-023-00623-z (PMC9948798; doi:10.1038/s41541-023-00623-z)
Supplement: Supplementary file 2 — REPORTING SUMMARY [file 41541_2023_623_MOESM2_ESM.pdf]

nature portfolio

Corresponding author(s): Zhou Xing

Last updated by author(s): Dec 7, 2022

Reporting Summary

Nature Portfolio wishes to improve the reproducibility of the work that we publish. This form provides structure for consistency and transparency in reporting. For further information on Nature Portfolio policies, see our Editorial Policies and the Editorial Policy Checklist.

Statistics

For all statistical analyses, confirm that the following items are present in the figure legend, table legend, main text, or Methods section.

n/a	Confirmed	
☐	☒	The exact sample size (*n*) for each experimental group/condition, given as a discrete number and unit of measurement
☐	☒	A statement on whether measurements were taken from distinct samples or whether the same sample was measured repeatedly
☐	☒	The statistical test(s) used AND whether they are one- or two-sided<br>*Only common tests should be described solely by name; describe more complex techniques in the Methods section.*
☐	☒	A description of all covariates tested
☐	☒	A description of any assumptions or corrections, such as tests of normality and adjustment for multiple comparisons
☐	☒	A full description of the statistical parameters including central tendency (e.g. means) or other basic estimates (e.g. regression coefficient) AND variation (e.g. standard deviation) or associated estimates of uncertainty (e.g. confidence intervals)
☒	☐	For null hypothesis testing, the test statistic (e.g. *F*, *t*, *r*) with confidence intervals, effect sizes, degrees of freedom and *P* value noted<br>*Give P values as exact values whenever suitable.*
☒	☐	For Bayesian analysis, information on the choice of priors and Markov chain Monte Carlo settings
☒	☐	For hierarchical and complex designs, identification of the appropriate level for tests and full reporting of outcomes
☒	☐	Estimates of effect sizes (e.g. Cohen's *d*, Pearson's *r*), indicating how they were calculated

Our web collection on statistics for biologists contains articles on many of the points above.

Software and code

Policy information about availability of computer code

Data collection	Immunostained cells were collected on a BD cytometer and assessed by FACSdiva (Version 8.0).<br>Western blot data was collected on the LI-COR Odyssey.<br>Images of representative micrographs were taken with a Zeiss Axio Imager 2 using Zen digital imaging software (Version 2.3).
Data analysis	Flow cytometry data was analyzed using FlowJo (Version 10.8.1).

For manuscripts utilizing custom algorithms or software that are central to the research but not yet described in published literature, software must be made available to editors and reviewers. We strongly encourage code deposition in a community repository (e.g. GitHub). See the Nature Portfolio guidelines for submitting code & software for further information.

Data

Policy information about availability of data

All manuscripts must include a data availability statement. This statement should provide the following information, where applicable:

- Accession codes, unique identifiers, or web links for publicly available datasets
- A description of any restrictions on data availability
- For clinical datasets or third party data, please ensure that the statement adheres to our policy

Data is available via the corresponding author upon reasonable request.

Human research participants

Policy information about studies involving human research participants and Sex and Gender in Research.

Reporting on sex and gender	N/A
Population characteristics	N/A
Recruitment	N/A
Ethics oversight	N/A

Note that full information on the approval of the study protocol must also be provided in the manuscript.

Field-specific reporting

Please select the one below that is the best fit for your research. If you are not sure, read the appropriate sections before making your selection.

☒ Life sciences ☐ Behavioural & social sciences ☐ Ecological, evolutionary & environmental sciences

For a reference copy of the document with all sections, see nature.com/documents/nr-reporting-summary-flat.pdf

Life sciences study design

All studies must disclose on these points even when the disclosure is negative.

Sample size	No statistical methods were used to pre-determine samples sizes.
Data exclusions	No data points were excluded from analysis.
Replication	Individual figure legends provide information regarding replication.
Randomization	Animals were assigned to experimental groups by random.
Blinding	Histological scoring and analysis were done in a blinded fashion by trained reseasearchers.

Reporting for specific materials, systems and methods

We require information from authors about some types of materials, experimental systems and methods used in many studies. Here, indicate whether each material, system or method listed is relevant to your study. If you are not sure if a list item applies to your research, read the appropriate section before selecting a response.

Materials & experimental systems

n/a	Involved in the study	
☐	☒	Antibodies
☐	☒	Eukaryotic cell lines
☒	☐	Palaeontology and archaeology
☐	☒	Animals and other organisms
☒	☐	Clinical data
☒	☐	Dual use research of concern

Methods

n/a	Involved in the study	
☒	☐	ChIP-seq
☐	☒	Flow cytometry
☒	☐	MRI-based neuroimaging

Antibodies

Antibodies used	Antibodies were purchased from BD biosciences unless otherwise specified. mCD45-AlexaFluor 700, hCD45-Pacific Blue, hCD3e-Qdot 605, hCD4-PerCP-Cy5.5, hCD8a-PE-Cy7, mCD3-V450, mCD8a–PE–Cy7, mCD4– APC–Cy7, mIFN-γ–APC, mTNFα-FITC, mIL2-PE, mCD45-APC-Cy7, mCD11b-PE-Cy7, mLy6C-Biotin, Streptavidin-QDot800 and mLy6G-BV605. h=Human reactive, m=Mouse reactive.
Validation	All antibodies are commercially available and validated by the manufacturer.

Eukaryotic cell lines

Policy information about cell lines and Sex and Gender in Research

Cell line source(s)	HEK-293, and A549
Authentication	Authentication from provider (ATCC).
Mycoplasma contamination	Testing was not performed.
Commonly misidentified lines (See ICLAC register)	N/A

Animals and other research organisms

Policy information about studies involving animals; ARRIVE guidelines recommended for reporting animal research, and Sex and Gender in Research

Laboratory animals	Wild type BALB/c mice were purchased from Charles River Laboratories (Wilmington, MA, USA). C3HeB/FeJ and NOD-Rag1null IL2rgnull (NRG) mice were purchased from Jackson Laboratory (Bar Harbor, ME, USA). Mice were housed in specific pathogen-free facility or the bio-safety level 3 facility with ad libitum access to food and water, 12 hour light cycle, 50-60% humidity and 20-25C room temperature at McMaster University, Hamilton, Canada.
Wild animals	N/A
Reporting on sex	Experiments with BALB/c or C3HeB/FeJ mice were performed with female mice. NRG mouse studies utilized male and female mice.
Field-collected samples	N/A
Ethics oversight	All animal experiments were reviewed and approved by the Animal Research Ethics Board at McMaster University. Experiments were carried out under the animal utilization protocol 210822.

Note that full information on the approval of the study protocol must also be provided in the manuscript.

Flow Cytometry

Plots

Confirm that:

☒ The axis labels state the marker and fluorochrome used (e.g. CD4-FITC).

☒ The axis scales are clearly visible. Include numbers along axes only for bottom left plot of group (a 'group' is an analysis of identical markers).

☒ All plots are contour plots with outliers or pseudocolor plots.

☒ A numerical value for number of cells or percentage (with statistics) is provided.

Methodology

Sample preparation	Animals were euthanized by exsanguination. In certain experiments, intravascular staining was carried out three minutes prior to sacrifice with anti-CD42.2 antibody. Cells from the bronchoalveolar lavage, lung tissue, and spleen were isolated. Red blood cells were lysed with an ACK lysis solution and cells were resuspended in complete RPMI media, as described in materials and methods section. Mononuclear cells were plated into 96 well U bottom plates at between 250,000-2,000,000 cells per well. Stimulations were performed for 4-6 hours with 5ug of each stimulant per well, with GolgiPlug (BD Biosciences).<br>Following stimulation, cells were centrifuged and pelleted, and staining was performed as described in the materials and methods section.
Instrument	Samples were acquired on the BD Fortessa.
Software	Flow cytometry data was collected with FACSDiva (version 8.0), and analysis was done on FLowJo (version 10.8.1).
Cell population abundance	Abundance of CD4 and CD8 T cells.
Gating strategy	Gating is described in the materials and methods section. Briefly, debris (FSC SSC) was gated out, followed singlet isolation. Viable cells were then gated and subsequent populations of-interest were gated accordingly.

☒ Tick this box to confirm that a figure exemplifying the gating strategy is provided in the Supplementary Information.
